# Understanding quantitative effects of anti-amyloid therapies on tau biomarkers and functional outcome. Insights from a comprehensive mechanistic quantitative systems pharmacology study

**DOI:** 10.3389/fphar.2026.1813290

**Published:** 2026-04-30

**Authors:** Hugo Geerts, Shaina M. Short, Athena Grant, Piet H. van der Graaf

**Affiliations:** 1 Certara Predictive Technologies, Applied Biosimulation, Radnor, PA, United States; 2 Leiden Academic Centre for Drug Research, Leiden, Netherlands; 3 Cincinnati Children’s Hospital Medical Center, Cincinnati, OH, United States

**Keywords:** amyloid biomarkers, amyloid load negativity, functional outcome, plasma tau biomarkers, responder profiles

## Abstract

**Introduction:**

Anti-amyloid antibodies have the potential to become the standard of care in Alzheimer’s Disease (AD) and large datasets from clinical trials allow the testing of predictive models on fluid biomarkers and functional outcomes. However, identifying an easily accessible biomarker to determine the time to switch to maintenance therapy, and identifying patient profiles with optimal cognitive benefit, are still unresolved issues in clinical practice.

**Methods:**

Predicted changes in monomers, oligomers, protofibrils and plaques were simulated using a well-validated Quantitative Systems Pharmacology model based on biophysical and biological assumptions of amyloid synthesis, aggregation and clearance. This model was combined with a previously calibrated computational neuronal network model of cognitive outcome in AD patients by introducing the effect of amyloid and tau oligomers on specific voltage- and ligand-gated ion channels, informed by preclinical studies.

**Results:**

The model accounted for 70% and 50% of the variance of clinically observed changes in plasma p-tau181 and Clinical Dementia Rating-Sum Of Boxes (CDR-SOB) respectively, in clinical trials of seven amyloid antibodies. We derived an antibody specific normalized decrease of plasma p-tau181 (−15% for donanemab, −45% for aducanumab and −75% for lecanemab) to determine trial duration for achieving central amyloid negativity. Using the concept of information processing bandwidth, the model suggests that anti-amyloid antibodies slow the cognitive worsening compared to placebo while at the same time lowering plasma p-tau181 levels by reducing neuronal firing. Finally, the model suggests that independently from the degree of amyloid reduction, the beneficial cognitive effect of treatment decreases with more advanced neuronal pathology and higher baseline tau-load. This provides a hypothesis for the impact of disease pathology and gender effect on functional outcomes with lecanemab and gantenerumab.

**Discussion:**

With further validation, this model has the capability to support optimization of clinical trial design for amyloid-tau combination therapy.

## 1 Background

With the approval of amyloid modulating biologics, focus has turned to optimizing access in clinical practice–however, current uptake is limited due to the expensive tests for measuring amyloid dynamics in patients during treatment. The gold-standard remains PET imaging of central amyloid load (defined as the total mass of soluble and insoluble Abeta species in the Interstitial Fluid), but a simpler and more accessible method to determine the time at which patients reach amyloid negativity and can be switched to maintenance therapy could help greatly in the uptake of these new therapeutics. While an earlier study suggested antibody-specific changes in CSF amyloid-beta 42 (Aβ42) can be used (Geerts et al., 2023a), a more accessible biomarker, such as plasma biomarkers that can report on dynamics of amyloid load during and after treatment, would be very helpful. In this regard, longitudinal observational studies have shown that changes in various forms of plasma p-tau181 can inform about the central amyloid load (Dark et al., 2024), and secondary readouts from Phase 3 studies with amyloid antibodies have documented a range of plasma p-tau181 or CSF p-tau181 changes at different time points after start of the treatment (McDade et al., 2022). However, a systematic study for their use in determining timing of central amyloid negativity is lacking.

More importantly, while changes in amyloid load as measured by standardized uptake value ratio (SUVr) grossly correlate with cognitive improvements over placebo, the contribution of changes in intermediate, but inaccessible, amyloid-beta (Abeta) species in driving cognitive outcome is unclear. This becomes important as a number of therapeutic antibodies with preferential activity against oligomers and more modest impact on SUVr are currently in clinical development with the rationale that they can result in a functional clinical benefit.

A previous publication used a semi-empirical approach to link amyloid, tau and cognitive functioning (Mazer et al., 2023). To address these two objectives more quantitatively, here we used a QSP model of amyloid aggregation that is well-validated using group average data on the clinical effect of six antibodies (Geerts et al., 2023b) and has recently been shown to generalize for other antibodies and other clinical scenarios (Geerts et al., 2023a). This model allows simulation of the effect of different antibodies with their appropriate dosing schedules on the dynamics of Abeta monomers, oligomers and protofibrils. Modeling the dynamics of these Abeta soluble oligomers is extremely interesting as new technological advances have demonstrated in a longitudinal study that Abeta oligomers peak early, well before tau pathology (Blömeke et al., 2023).

Using regression analysis, we can then identify the contribution of these inaccessible Abeta species to experimentally observed changes in p-tau181 and cognition in actual clinical trials and compare to the correlation with observed changes in a readily accessible biomarker such as amyloid SUVr.

The purpose of this report is to provide a quantitative and biologically plausible mechanistic relationship between changes in various central amyloid species and reduction in p-tau181 fluid biomarkers and cognitive performance on the other hand.

In particular, we explore whether the plasma p-tau181 biomarker could be used to indicate the time at which amyloid load drops below the threshold for positivity and how this biomarker changes during the maintenance stage after treatment halt. Using a virtual patient trial, we will investigate the robustness of this plasma biomarker for monitoring central amyloid load for a large number of different individual patient baselines and characteristics.

Levels of secreted tau molecules, as reflected in plasma or CSF p-tau181 are driven by changes in neuronal firing (Yamada et al., 2014). As amyloid pathology in late mild cognitive impairment (MCI) and early prodromal AD is associated with hyperactivity (Wang et al., 2009; 2013) due to its effects on glutamate, nAChR and K+ conductance (Fernandez-Perez et al., 2021; George et al., 2021; Olah et al., 2022; Ping et al., 2015; Ren S. Q. et al., 2018), it makes sense that reducing amyloid burden would lower tau secretion and therefore observed levels in plasma and CSF p-tau181. This provides a qualitative mechanistic rationale for the link between amyloid and tau changes.

To derive a more quantitative relationship between amyloid changes and plasma p-tau181 changes for clinical practice, we use an actual computational neuroscience model (Geerts et al., 2018; Geerts and Spiros, 2020a; Geerts and Spiros, 2020b; Roberts et al., 2012) that captures the effect of amyloid pathology on neuronal firing. Next, we generate a quantitative relationship between neuronal firing and tau secretion, based on preclinical experimental data (Yamada et al., 2014). Most importantly, this will allow us to derive a quantitative relationship between changes in various Abeta species, impact on neuronal firing and tau secretion and ultimately fluid p-tau181 biomarkers for a large number of individual patient baselines and characteristics in order to determine the timing at which the central amyloid load reaches negativity.

Post-hoc analyses of anti-amyloid antibodies have highlighted the impact of baseline tau pathology, not on amyloid biomarkers but most importantly on the functional clinical outcomes (Sims et al., 2023). Also, a recent study suggests that lecanemab as compared to gantenerumab and aducanumab has a higher affinity for small diffusible Abeta species (Fertan et al., 2025) that are prominent in early disease, possibly leading to a more robust functional outcome. We address these questions using a computational neuroscience model that has been calibrated with ADAS-Cog scores (Roberts et al., 2012) after implementing the documented effects of these Abeta and tau oligomers on voltage- and ligand gated ion channels. This would allow us to explore the effect of amyloid therapy on neuronal firing in appropriate networks driving functional cognitive outcome.

Finally, such a combined mechanistic Amyloid-Tau QSP model linking biomarker and functional outcomes could inform future clinical trial design of combination therapies (Boxer and Sperling, 2023).

## 2 Methods

### 2.1 Amyloid aggregation model

Calculation of intermediate Aβ42 species such as ISF monomer, oligomer (dimer to 16-mer), protofibril (17–23-mer) and plaques (24-mer and above) is performed using the amyloid aggregation QSP model (Geerts et al., 2023b). Basically, this model simulates (1) the synthesis of Aβ40 and Aβ42, (2) the breakdown of monomers through IDE and neprilysin, (3) the forward aggregation of monomers into higher-order aggregates, (4) the backward generation of monomers from higher-order aggregates, (5) microglia-dependent amyloid clearance, and (6) the distribution of soluble monomers from the brain into the CSF and plasma. Drug exposure of amyloid antibodies is modeled using a minimal PBPK model (Bloomingdale et al., 2021) and the binding of Immunoglobulin G1 (IgG1) isotypes to the Fc gamma receptor (FcγR) on microglia triggers a phenotypic switch in microglia resulting in higher clearance of amyloid species. Extensive documentation on equations and parameters can be found in the Supplementary Material (Geerts et al., 2023a; Geerts et al., 2023b) The model is well calibrated using both natural history data and the pharmacodynamic effect of six antibodies on amyloid biomarkers and has recently been applied to explore practical challenges in clinical practice (Geerts et al., 2023a). The model runs in QSPDesigner (Matthews et al., 2023) and ODE solver is based on backward differentiation formula (BDF) with an absolute tolerance of 10E-15. A typical run on a laptop CPU takes about 20 min to simulate 16,000 time points.

### 2.2 Clinical data on p-tau181 biomarker

To collect the clinical data on changes in plasma p-tau181 or CSF p-tau181 biomarkers and on cognitive changes (CDR-SOB), we used Certara’s CODEX database (Certara CODEx and Clinical Outcomes Databases|Quantifying data) from donanemab, lecanemab, aducanumab, gantenerumab, solanezumab, crenezumab and bapineuzumab clinical trials. We identified 35 datapoints of p-tau181 changes and 36 datapoints for cognitive changes *versus* placebo at different time points, different doses and titration schedules.

Careful analysis of the reported changes with donanemab, lecanemab and aducanumab suggests that lecanemab has the greatest effect on reduction of plasma or CSF p-tau181. It is of interest to more closely examine the effect of lecanameb. In the CLARITY Phase 3 trial (van Dyck et al., 2023), a decrease of 18 pg/mL for CSF p-tau181 and a plasma decrease of 0.6 pg/mL was reported. Given that baseline CSF p-tau181 is around 30 pg/mL in Braak stage I and II, rising to 50 pg/mL in Braak stage III and above (Kurihara et al., 2024), the change in CSF p-tau181 is well above 50%. On the other hand, a 0.6 pg/mL reduction (only a 10% reduction from baseline) in plasma p-tau181 as reported in VanDyck’s Supplementary Figure SH is well below detection limit (technical fiche Quanterix Simoa, suggesting that the y-axis label in this figure (p-tau changes after lecanemab treatment) might be more correctly interpreted as the normalized decrease in plasma p-tau181. Such a decrease is more in line with the reported 12.3 pg/mL decrease for CSF p-tau181 in the adaptive Phase 2 trial (Swanson et al., 2021). For donanemab’s TRAILBLAZER trial, a −0.12 log10 value corresponding to a 10^-0.12 or 0.75 was mentioned, corresponding to a 25% decrease in CSF p-tau 217 (Pontecorvo et al., 2022). All values are provided in Supplementary Table S1.

### 2.3 Multivariate regression for plasma p-tau181 changes and cognitive outcome

We simulated the changes in intermediate Abeta in our amyloid aggregation model, such as ISF Aβ42 monomers, Aβ42 oligomers, Aβ42 protofibrils and Aβ42 plaques normalized to baseline values for the different clinical trial designs.

Statistical analysis of linear and multivariate regression between the simulated changes in Abeta species and the observed outcomes on plasma p-tau181 and CDR SOB is executed using the Statistical Analysis Package of Excel software.

### 2.4 Time to reach amyloid negativity

For relating the fluid p-tau181 levels to the treatment duration for achieving central amyloid negativity, we simulated clinical trials of aducanumab, lecanemab, and donanemab at different starting baseline amyloid levels. Using the formula from the multivariate analysis, we calculated the corresponding plasma p-tau181 levels from the trajectory of the four different Abeta species. We determined the duration to reach a central amyloid load of 25 CL and calculated a value for plasma p-tau181 at the time of amyloid negativity for each of the antibodies and as a function of amyloid baseline for each virtual patient in the simulated trial.

### 2.5 Virtual patient trial

We generated a virtual patient population (n = 1,000), with about 75% APOE4+ subjects first for the longitudinal unmedicated condition to determine the natural trajectory of amyloid aggregation by sampling parameters associated with biological processes from a Gaussian distribution with a CV of 10%–30% depending upon the individual parameter (Geerts et al., 2023a). This reflects grossly the distribution of amyloid biomarkers observed in the Phase 3 studies.

Biological processes unique for individual patients include synthesis and degradation of Aβ monomers, forward and backward rate constants of primary nucleation for higher-order aggregates, enhanced forward aggregation due to secondary nucleation, breakdown of protofibrils into smaller aggregates and uptake of various Abeta species by microglia cells. Additionally, variability of drug uptake at the Blood-Brain Barrier and the phenotypic switch from low to high phagocytosing phenotype after binding of the antibody to the Fc-gamma receptor (FcγR) on microglia cells can be observed.

We identified the age at which the SUVr amyloid exceeded 1.30 or 47 centiloids (Age*). We subsequently simulated an 18-month trial for donanemab, lecanemab and aducanumab for the same patient where the start of the trial was delayed according to the following relationship, assuming an average delay of 6 years between diagnosis and treatment start.

$$\text{Start}\_\text{Trial }\left(\text{in years}\right)=\text{Age}*+2+8*\text{RND }\left[0,1\right]$$

Where RND (random) is sampled from a uniform distribution between 0 and 1. The average age of patients at the start of treatment was 74.4 ± 4.49 years (range 61–90 years), while the average baseline amyloid load was 120 ± 20 CL (range 98–180). Gender was implemented using an average body weight of 55 kg for females and 79 kg for males and was evenly distributed (50/50).

Readouts of interest include dynamics of free monomeric Aβ42, oligomeric Aβ42, protofibril Aβ42, plaque Aβ42 and the clinical outcomes of CSF Aβ42, SUVr. This allows us to compare the effect of the three drugs on exactly the same patient population.

### 2.6 Mechanistic model of amyloid and tau effects on cognitive readouts

Based on the premise that cognitive outcome is driven by properties of neuronal firing in well-defined neuronal circuits, we simulated realistic firing patterns using a biophysical representation of the opening and closing of relevant voltage- and ligand-gated ion channels (Hodgkin and Huxley, 1952).

This computational neuroscience model, calibrated to longitudinal and interventional clinical data on cognitive outcome, such as ADAS-Cog has been published before (Roberts et al., 2012). Basically, the computer model generates biophysically realistic action potential dynamics in a cortical microcolumn with 80 pyramidal cells and 40 GABA inhibitory neurons, leading to an appropriate balance of excitation and inhibition. At a specific time, a short 50 msec stimulus gives rise to a continuous firing of a subset of pyramidal neurons over a number of seconds. This time span is reminiscent of a working memory trace stability and is strongly correlated to an actual N-back working memory task (Geerts et al., 2013). Although the interventional studies used for calibration mostly used short-term symptomatic treatment affecting neuronal firing, the model also takes into account a progressive neuronal and synapse loss, mostly due to increasing tau pathology based on long-term longitudinal studies.

Aβ40 and Aβ42 monomers have a differential impact on glutamate and alpha 7 nicotinic acetylcholine receptor (α7 nAChR) neurotransmission, whereby the long peptide Aβ42 has a monotonic inhibitory effect on glutamate tone, while Aβ40 shows an inverse U-shape dose-response (Wang et al., 2013). The model, based on these assumptions, was able to explain three clinical datasets on pharmacological challenges in Abeta positive and Abeta negative MCI patients (Doraiswamy et al., 2014; Lim et al., 2015) and predicted the transient functional worsening of beta-amyloid precursor protein cleaving enzyme 1 or BACE-inhibitors.

We further implemented the effect of soluble Abeta oligomer species on neuronal firing based on the following effects of oligomeric Abeta reported in preclinical experiments: (1) decrease in potassium conductance, gK (Ping et al., 2015), (2) increase in α7 nAChRconductance (George et al., 2021), (3) increase in pyramidal excitatory AMPA receptor (Fernandez-Perez et al., 2021), (4) increases in GABAergic conductance on inhibitory neurons likely due to decreases in interneuron gK (Ren S. Q. et al., 2018) and the voltage gated potassium Kv3 channel in parvalbumin cortical interneurons (Olah et al., 2022). These combined interventions lead to a higher firing frequency with increasing levels of Abeta species (Minkeviciene et al., 2009; Tamagnini et al., 2015; Varghese et al., 2010).

Soluble tau oligomers have been documented to increase the width of the action potential (Hill et al., 2019; 2021) and affect the speed of action potential propagation (Sohn et al., 2019). This effect was driven by a direct interaction of tau oligomers with voltage-gated potassium K^+^ and sodium Na^+^ channels. To fully explain the observed quantitative effects on action potential propagation as observed in multielectrode array (MEA) studies of human induced pluripotent stem cells (hIPSCs) from frontal temporal dementia (FTD) donors (Sohn et al., 2019), the effect of tau oligomers was implemented using a maximal 10% reduction of Na channel conductance, and a maximal reduction of 10% for voltage-gated K^+^ channels in the whole neuron. This was then extrapolated to our Alzheimer’s Disease QSP model based on the assumption that the V337M FTD mutation had the same impact on voltage-gated ion channels.

We implemented the effects of Abeta soluble oligomers as follows. Calculating levels of soluble Abeta oligomers from the observed SUVR changes (between 1 and 2), we arrive at

$${\text{gAMPA}}^{e-e}={\text{gAMPA}}^{e-e}{}_{\text{base}}*\left(1+\left({\text{SUVR}}_{\text{amy}}-1\right)/5\right)$$

$${\text{gGABA}}^{e-i}={\text{gGABA}}^{e-i}{}_{\text{base}}*\;\left(1-\left({\text{SUVR}}_{\text{amy}}-1\right)/5\right)$$

$${\text{gK}}^{\text{pyr}}={\text{gK}}^{\text{pyr}}{}_{\text{base}}*\left(1-\left({\text{SUVR}}_{\text{amy}}-1\right)/10\right)$$

$${\text{gK}}^{\mathrm{int}}={\text{gK}}^{\mathrm{int}}{}_{\text{base}}*\left(1+\left({\text{SUVR}}_{\text{amy}}-1\right)/5\right)$$

where superscripts int and pyr refer to inhibitory and pyramidal cells, respectively and superscripts e-I and e-e refer to excitatory-inhibitory and excitatory-excitatory projects respectively. The subscripts “base” refers to the baseline value of the conductance (i.e., in the absence of any amyloid and tau pathology).

The effects of tau pathology on the K+ and Na+ channels are implemented as follows with Tau PET SUVR_tau_ between 1 and 3

$$\text{gK}={\text{gK}}_{{}_{\text{base}}}*\left(1-\left({\text{SUVR}}_{\text{tau}}-1\right)/20\right)$$

gNa = gNa _base_ *(1-(SUVR_tau_−1)/20) with Na-channels only considered in the Axonal Initial Segment (AIS).

These values were chosen to align best with the clinical changes

The values of the above defined coupling constants between amyloid and tau SUVR and the impact of various neuronal processes is determined from observations on plasma p-tau changes as outlined in Methods Section 7.

This computational neuroscience QSP model runs in the open-source NEURON software (Hines and Carnevale, 2001) and uses a time-resolution of 0.5 msec. Calculation of the relevant readouts (see below) is done over 30 episodes of 12 s each (24,000 time points). A single run takes about 20 min laptop CPU time; hyperthreading allowd to run 12–16 simulations in parallel.

An interesting readout for cognitive performance of the QSP model is the information bandwidth based on the Shannon entropy (Strong et al., 1998). This is based on the observation that not the firing frequency, but the inter-spike interval variability, is associated with better cognitive performance as it can be encoded with a larger number of bits in a binary word. Here we project the action potential of all pyramidal cells and interneurons on one time axis and determine the Shannon entropy of the inter-spike interval distribution. The detailed implementation is described in the Supplementary Material (Supplementary Section S3). Both high and low frequency firing have a more limited variability and therefore can carry less information. This approach captures the reduced information capacity of a hyperactive state, as observed in high-amyloid load MCI patients.

Disease state was implemented using a state-dependent elimination of neurons and synapses. We define four prodromal (MCI-like) states, MCI1 to MCI4 with increasing fractional loss of synapses (1%–4%) and neurons (1%–4%) in our computational neuroscience network. This is accompanied by a compensatory increase in N. Basalis Choline acetyl transferase (ChAT) activity boosting acetylcholine (ACh) levels (Ikonomovic et al., 2007). At AD0, the onset of Alzheimer symptoms, from previous calibration (Roberts et al., 2012), the fraction of neurons and synapses lost is 5%, increasing to 6.9% and 5.29% at AD6 and 8.1% and 5.48% at AD12, respectively. Note that an additional synapse loss is implemented after the neuron loss, adding to the synapses that are lost because of neuron elimination. Due to the increased N Basalis pathology in Alzheimer’s Disease, the compensatory upregulation of Ach synthesis as observed in the MCI case is lost, we therefore assume a 30% decrease in Ach levels for the AD0, AD6 and AD12 states (Roberts et al., 2012).

### 2.7 Relationship between neuronal firing and tau secretion

Presynaptic release of tau proteins into the synaptic cleft has been suggested to be mediated by the synaptic vesicle release machinery (Tracy et al., 2022), and therefore can be dependent upon the neuronal firing activity of the afferent neuron. As amyloid antibodies decrease neuronal firing, this leads to decreased secretion of tau and p-tau181 which can be detected in CSF and plasma as a decrease in tau biomarker levels.

To determine the quantitative relationship between firing and tau release, we started from preclinical data in wild-type and D280K transgenic mouse with a variety of pharmacological interventions (picrotoxin, NMDA, the mGLUR2 modulator LY341495 and TTX) to modulate the *in vivo* cortical firing frequency (Yamada et al., 2014). We simulated the same pharmacological interventions in the NEURON computational neuroscience cortical model and derived a relationship between the firing frequency and the corresponding experimentally determined tau increase.

Using the average decrease of plasma p-tau181 from the lecanemab trials, we can derive the corresponding decrease in neuronal firing, which would then define the coupling factor between oligomer concentration and effect on neuronal firing as described in Methods Section 6.

### 2.8 Relationship between ADAS-Cog and CDR-SOB

As most of the anti-amyloid therapies used CDR-SOB as primary outcome, we wanted to derive a relationship with ADAS-Cog so that we could use the previously calibrated ADAS-Cog model (Roberts et al., 2012). We used Certara’s CODEX database and identified 298 cases where both ADAS-Cog and CDR-SOB were reported.

This ultimately results in the formula CDR-SOB = 3.5–0.02*(InfCon-415). The derivation of the information content, InfCon, was based on the statistics of inter-spike intervals and is explained in Supplementary Section S8.

## 3 Results

### 3.1 Regression analysis of QSP model outcomes with clinical observation of p-tau181 changes

For the 35 clinical data-points on plasma p-tau181 changes with amyloid antibody treatment, we simulated the trajectories of intermediate inaccessible biomarker dynamics of Abeta species (monomers, oligomers, protofibrils and plaques) using the appropriate clinical trial design for each of the studies.

**FIGURE 1:**
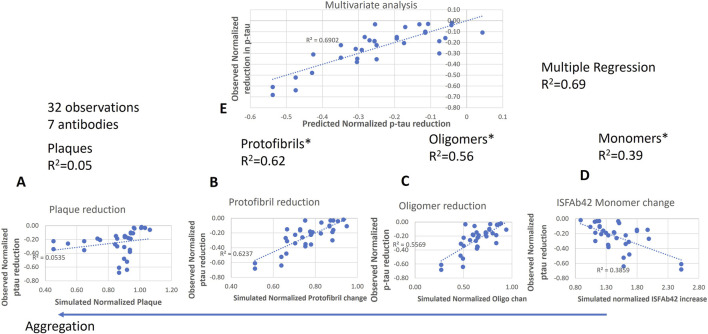
Correlation analysis between 32 (drug-dose-time combinations) simulated changes of plasma p-tau181 normalized to their baseline as a function of simulated ISF plaques **(A)**, protofibrils **(B)**, oligomers **(C)** and monomers **(D)** dynamics of Aβ42 in the QSP model for the same AD patient treated with seven different antibodies with the dosing regimen from the respective clinical trials, *versus* reported changes in biomarker plasma p-tau181. Correlation is weak for plaque changes (left) and much greater by changes in soluble Abeta species. Multivariate regression analysis (top) suggests that the model can explain 69% of the variance **(E)**. We use the coefficients of this multivariate regression analysis to calculate the estimated p-tau181 dynamics from the QSP model outcomes in the virtual patient trial simulation.

Figure 1 shows that correlation between simulated changes and observed changes in p-tau181 is the highest for changes in protofibril Aβ42 (*r* ^2^ = 0.62), oligomeric Aβ42 (*r* ^2^ = 0.56), followed by ISF monomeric Aβ42 (*r* ^2^ = 0.39) and finally plaque Aβ42 (*r* ^2^ = 0.05). This suggests that changes in p-tau181 are driven by more soluble Abeta species in the aggregation cascade. The correlation between experimental changes in plasma p-tau181 and predicted changes in SUVr in the QSP model can account for only 25% of variability.

A multivariate regression analysis using these simulated model outcomes can account for 69% of the variability of p-tau181 changes (p-value = 0.00029). Using this information, we can derive the equation below with the appropriate contributions of each Abeta species. The following formula captured almost as good the change in plasma p-tau181 biomarker explaining 66% of the variability.

$$\text{Ptau}\left(t\right)/\text{Ptau}\left(0\right)=C_{1}+C_{2}\left[C_{3}+C_{4}\left(1-\left({\text{Ab}}_{\text{mono}}*\left(t\right)–C_{5}\right)/C_{5}\right)+C_{6}*{\text{Ab}}_{\text{oligo}}*t+C_{7}*{\text{Ab}}_{\text{proto}}*\left(t\right)+C_{8}*{\text{Ab}}_{\text{plaque}}*\left(t\right)\right]$$

where Ab_X_*(t) = Ab_X_(t)/Ab_X_ (0) with X = mono, oligo, proto, plaque.

And corresponding values C1 (0.812), C2 (0.53), C3 (−1.93), C4 (0.23), C5 (30), C6 (0.43), C7 (1.45) and C8 (−0.04).

### 3.2 Simulating dynamics of p-tau181 biomarker in virtual patient trial

When applying this formula to the simulated individual Abeta species outcomes, we can calculate the decrease in p-tau181 at the time that the SUVr reaches negativity (a value of 25 CL) for each of the 1,000 virtual patients treated with the different antibodies.

**FIGURE 2:**
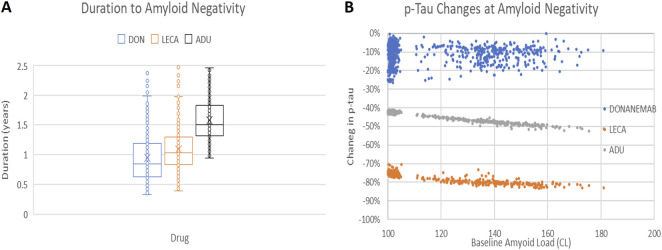
**(A)** Duration to reach amyloid negativity for 1,000 virtual APOE4+ patients treated with donanemab, lecanemab and aducanumab. As expected, donanemab achieves amyloid negativity the fastest, followed by lecanemab and aducanumab, reflecting both the affinities of the antibody for the different Abeta species, as well as the titration schedule. **(B)** Relationship between baseline amyloid level and estimated changes in plasma p-tau181 at amyloid negativity for 1,000 virtual patients treated with donanemab, lecanemab and aducanumab. The outcome suggests that decrease in the range of 70% and 40% in plasma p-tau181 levels can be used to determine the time to reach amyloid negativity with lecanemab and aducanumab, respectively. In contrast, it is more difficult to define a threshold for donanemab.

Figure 2 shows the simulated plasma p-tau181 changes for each of the virtual patients at the time of reaching amyloid negativity for that patient baseline amyloid load. The outcomes suggest a small decrease (10%–20% effect size) in p-tau181 for donanemab. In contrast, both aducanumab (41%) and lecanemab treatment (77%) lead to a substantial change in p-tau. This suggests that there is not “one size fits all” for the use of a plasma p-tau threshold for determining the time of amyloid negativity but that it is dependent upon the pharmacology of the antibody. Similar results have been obtained for a virtual patient population consisting of nonAPOE4 subjects. The most convenient way for the patients to have plasma samples for p-tau181 taken is at the infusion times (i.e., Q4W or Q2W). Careful analysis of the time at which amyloid negativity is reached (see Supplementary Material Section S2) suggests that it is worthwhile to start analyzing these samples from six and 12 months onwards for lecanemab and aducanumab respectively.

### 3.3 Mechanistic model for amyloid-tau relationship

#### 3.3.1 Relating cortical firing to tau secretion

While the previous results were derived from empirical associations between amyloid modeling outcomes and clinical findings, in this section we will explore a more mechanistic understanding of the changes in plasma p-tau181. Tau secretion has been documented to be driven by synaptic activity (Yamada et al., 2014); to this end we simulated preclinical experiments, to the extent that average extracellular tau level *in vivo* ranges from 0.7 to about 1 nM in wild-type mice with 1%–2% of seed-competent tau and 4%–6% of tau in exosomes (Wang et al., 2017).

In the Yamada experiment, wild-type mice were treated with seven pharmacological interventions (see Supplementary Material Section S5) that increased ISF tau levels in a range between 0% and 200%. We ran these same pharmacological interventions in 3 different QSP models (corresponding to a heathy case, a “MCI” type case with minimal loss of synapses and neurons and compensatory increase of Ach and an “AD” case with more pronounced loss of synapses and neurons in addition to a hypo-cholinergic state, see also Methods Section 6) to identify the changes in firing rates and correlated these with the observed increase in ISF tau. A weighted slope between fractional increase in ISF tau and firing was determined to be 12.92 (i.e., for each percent increase in neuronal firing, a 12.9% increase in ISF tau was predicted). For the clinically observed decreases in plasma p-tau181 (see Methods Section 2), this would correspond to a range between 4% (aducanumab) and 6% change (lecanemab) in firing frequency.

#### 3.3.2 Modeling the effect of soluble Abeta species changes on functional outcomes

In general, *post hoc* analyses from the clinical trials with anti-amyloid antibodies suggest that the functional cognitive worsening was smaller for earlier stages of pathology and lower baseline tau levels (Christopher et al., 2023; Bateman et al., 2023).

**FIGURE 3:**
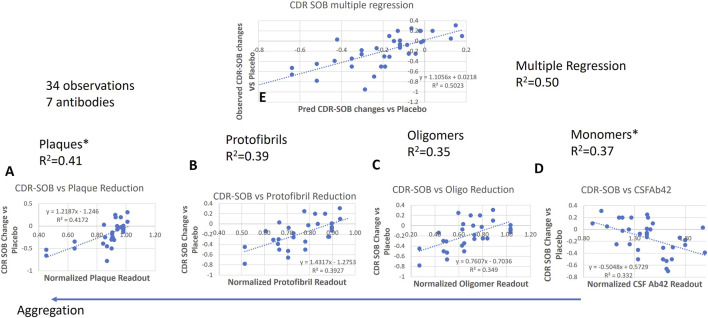
Correlation analysis between 32 (drug-dose-time combinations) simulated ISF plaques **(A)**, protofibrils **(B)**, oligomers **(C)** and monomers **(D)** dynamics of Aβ42 in the QSP model for the same AD patient treated with 7 different antibodies with the dosing regimen from the respective clinical trials, *versus* 34 reported changes in CDR-SOB from baseline of active treatment *versus* placebo (negative values refer to slower cognitive worsening). Correlation is strongest for plaque changes (left) but also quite elevated for changes in soluble Abeta species. Multivariate regression analysis **(E)** suggests that the model can explain almost 50% of the variance.

Given that our analysis of functional readout on CDR-SOB in the clinical trials was dependent upon changes in all Abeta species (Figure 3), we next investigated whether implementation of the documented biological effects of soluble Abeta species (defined as monomers, oligomers and protofibrils) on neuronal activity could reproduce the observed effects on functional outcomes.

First, in the absence of any tau pathology, after implementing specific effects of soluble Abeta42 species (Methods Section 6), the model predicts increased firing with a higher level of soluble Abeta42 species as expected (Supplementary Figures S2–S5 in Supplementary Material Section S4; Supplementary Figure S5). The firing frequency increases proportionally with the level of soluble Abeta42 species with baseline levels depending upon the disease state (lower firing frequency with more advanced AD pathology), while the information processing capacity has a more complex relationship.

We derive a quantitative relationship between changes in the different affected processes (NMDA, AMPA, α7 nAChR and gK) and soluble Abeta species from the clinical observations on the reduction in neuronal firing corresponding to the observed clinical decrease in plasma p-tau181. We define amyloid oligomer strength as the relation between absolute amyloid oligomer levels and effects on electrophysiological properties. As demonstrated in Supplementary Material Section S5, the reduction in neuronal firing can be achieved for donanemab by a decrease of amyloid oligomer strength of 2% (for an early AD subject) to 8% (for an early MCI subject). Conversely, for lecanemab, this can be achieved by a 4% decrease in the level of soluble Abeta42 species (mild AD subject) to a 10% decrease (early MCI subject) (see Supplementary Table S3).

#### 3.3.3 Modeling the effect of soluble Abeta species changes in different tau environments on CDR-SOB predictions

Once we quantified the impact of soluble Abeta species on neuronal firing, we then added the effect of tau pathology on voltage gated ion channels (Methods Section 8). However, unlike the amyloid case, we do not have the same information for quantifying the effect of pathological tau changes on neuronal firing.

At any specific level of tau pathology, increasing soluble Abeta species increases firing in a disease state dependent way, as outlined above for the no tau case. We arbitrarily divided tau oligomer levels in 8 units from Tau0 – no detectable tau level as in the healthy control case–to Tau8 – associated with Braak stage VI. Because increasing tau pathology by itself reduces firing frequency due to its effect on Na-conductances, the relative increase triggered by the same increase in Abeta oligomer strength is greater for higher tau pathologies. For example, the increase in firing frequency for Tau0 *versus* Tau8 level is 5.2% vs. 6.4% for the MCI1 state, 3% vs. 11% for the MCI4 state and 22.1% vs. 26.6% for the AD12 state, respectively.

Similarly, at any level of baseline tau pathology, the predicted CDR-SOB features an inverse U-shape dose-response for the level of soluble Abeta42 species as observed in the no tau case. As expected, increasing tau pathology in general decreases cognitive performance as measured by the information content (see Supplementary Figure S7). In addition, increasing disease state shifts the peak of best cognitive performance (highest information capacity) towards higher Abeta oligomer values. When averaging over all tau pathology levels, the best cognitive performance at MCI1 is at an Abeta oligomer strength of zero, at 5.5% for MCI3, at 10% for AD0, and finally 12.5% for AD12. The level of soluble Abeta42 species at which optimal cognitive performance happens is relatively independent of tau level (see Supplementary Figure S7).

The QSP model was previously calibrated using 28 datapoints from ADAS-Cog readouts in clinical trials for symptomatic therapies (Roberts et al., 2012). We used the CODEX database of more than 100 Alzheimer disease trials for identifying clinical datapoints for both CDR-SOB and ADAS-Cog to derive a relationship between these two functional scales (Supplementary Material Section S8; Supplementary Figure S8) that would convert information content readout from the QSP model to actual CDR-SOB values.

#### 3.3.4 Simulating CDR-SOB changes with amyloid antibodies

**FIGURE 4:**
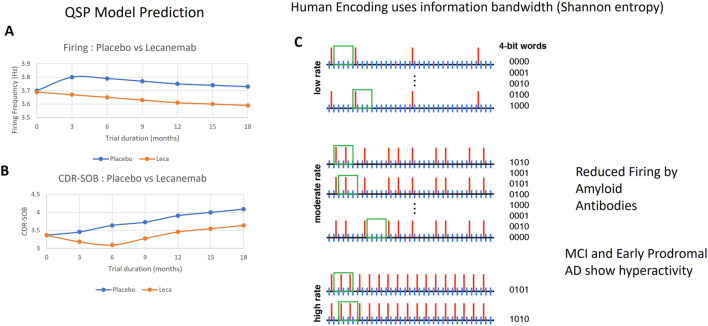
Simulation of a typical trajectory on average firing frequency **(A)** and cognitive outcome **(B)** for a generic patient treated with placebo and lecanemab. While the simulation shows that relative to placebo, lecanemab decreases neuronal firing, leading to a reduction in plasma tau level; at the same time lecanemab has a smaller cognitive deterioration than placebo (0.4 points vs. 0.8 points on the CDR-SOB scale). **(C)** graphically shows that by reducing the neuronal firing, lecanemab drives the system in an intermediate firing frequency regimen with a greater variability of interspike interval statistics, suggesting improved cognitive performance.

Once we had simulated CDR-SOB as a function of amyloid, tau oligomers, and disease state, we then simulated the trajectories of patients in the placebo and active treatment arms in anti-amyloid clinical trials under varying scenarios. The model predicts that increased soluble Abeta42 species in the placebo arm increases the average firing frequency. The clinical observations that placebo patients worsened around 1.6 points on the CDR-SOB over the span of 18 months (Sims et al., 2023) as a consequence of oligomer and plaque amyloid increase provide a calibration for the simulated firing increase. Figure 4 illustrates the effects of reducing soluble Abeta42 species with lecanemab vs. the placebo arm in a simulation of the 18-month trials, suggesting the simulated firing frequency and cognitive performance moving in opposing direction for the two treatment arms. The figure also graphically depicts the concept of information content, suggesting that reducing soluble Aβ42 oligomers by lecanemab reduces the firing frequency, resulting in larger information bandwidth (number of words or information to be carried by that spike train).

**FIGURE 5:**
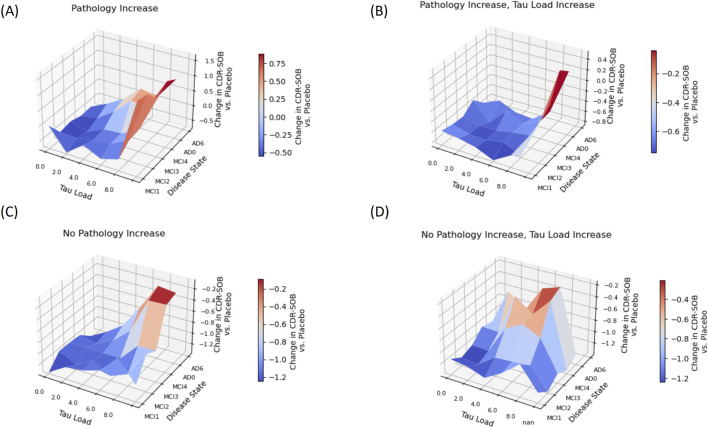
Surface plots demonstrating the effect of baseline disease state (referred to as MCI1 to AD6 or MMSE between 30 and 22) and baseline tau load (0–8 or Tau PET SUVr between 1 and 3) on the functional improvement in CDR-SOB from baseline relative to the placebo values (negative values mean a slower worsening with treatment) for four different progression conditions. In **(A)**, pathology increases by 1 unit, in **(B)** both pathology and tau load increase by 1 unit, in **(C)** neither pathology nor tau load increases, in **(D)** only tau load increases 1 unit over the duration of the trial. Blue colors are associated with treatment being more favorable than placebo while the inverse is true for red colors. Most of the blue colors are situated in the lower left, suggesting that amyloid antibodies have a better functional outcome in baseline conditions of lower pathology and tau load, despite the same change in amyloid biomarkers.

We next investigated the trajectory of different individual cases as a function of possible changes in disease state (i.e., increasing number of synapses and neurons lost) and progression of tau pathology (Figure 5).

As we do not have any information about the progression of the disease or tau load from the clinical trials, we simulated a variety of scenarios. When the patients remain in the same disease state and do not increase their tau load (Figure 5C), the average CDR-SOB difference between treatment and placebo is −0.46, −0.33, and 0.87 for the low disease/low baseline tau, intermediate disease state/moderate baseline tau, and advanced disease state/high baseline tau level respectively (negative values indicating better performance). Other analyses showing the same trend are reported in Supplementary Material Section S7.

Figure 5 shows the difference between treated patients and placebo for other baseline scenarios where pathology is increased by one unit (Figure 5A), where tau load is increased by 1 unit (Figure 5D) and where both pathology and tau load are increased by 1 unit (Figure 5B) over the time-course of the 18-month clinical trial. Blue colors are associated with less progression of CDR-SOB compared to placebo and red colors identify baseline conditions where placebo functional trajectory outperforms treated patients. The figures demonstrate that the blue colors tend to dominate the left lower part of the plotted surfaces, suggesting that the greatest beneficial effects of reducing soluble oligomers with amyloid therapies are in subjects with low pathology (MCI1-MCI3) and relatively low tau load (Tau0-Tau2).

## 4 Discussion

This paper reports on the development and applications of a combined amyloid-tau-functional outcome mechanistic QSP model, informed by the biology of amyloid and tau and their effects on voltage- and ligand-gated ion channels, leading to changes in neuronal firing dynamics. We use the concept of virtual patients to extend the clinical observations from the group level data to inform about outcomes in a more real-world clinical practice environment.

The model suggests that changes in plasma p-tau181 could be used to inform the duration of anti-amyloid treatment to reach central amyloid negativity at the individual patient level. Outcomes suggest that, at the time for reaching amyloid negativity, donanemab, aducanumab and lecanemab do change plasma p-tau181 to about 15%–20%, 40%–50%, and 70%–80% respectively. The decrease predicted for donanemab might be at the limit of detectability. Here we focused on p-tau181 based on the majority of clinical data for this epitope. In principle, other p-tau epitopes could be used after appropriate calibration.

This outcome is driven by the observation that tau secretion is neuronal activity dependent. While this has been demonstrated directly in in vitro cell cultures (Pooler et al., 2013; Croft et al., 2017; Bilousova et al., 2016) and animal models using pharmacological interventions (Yamada et al., 2014) and optogenetics (Wu et al., 2016), there is also evidence from MAPT gene mutation V337M Tau hIPSC that tau interacts with proteins of synaptic vesicle recycling and fusion and could use synaptic vesicle release (Tracy et al., 2022). Indirect evidence in aged humans includes the observations from the Harvard Ageing Brain Study that tau deposits using the radiotracer AV-1451 are associated with the projection regions of hot spots in BOLDfMRI, for example, hippocampal BOLDfMRI related to inferior temporal tau deposition (Hanseeuw et al., 2019; Huijbers et al., 2019).

This would also suggest that tau levels might also be affected by the various comedications and common genotype variants that affect neuronal firing, ranging from reductions (benzodiazepines) to increases (cholinergic standard-of-care). It also suggests that changes in this fluid tau biomarker are a direct and acute effect of amyloid therapy on neuronal firing and not necessarily indicate an effect on tau pathology progression.

The reduction of the uptake of misfolded tau protein in the afferent neuron by anti-amyloid therapies could over time lead to less formation of soluble intracellular tau oligomers and therefore reduction of tau pathology, although timelines are likely much longer than the typical duration of an amyloid antibody treatment. This also opens the intriguing possibility that drugs aimed at soluble Abeta oligomers, such as lecanemab, over time might have a greater impact on tau pathology, as they have a greater cumulative effect on neuronal firing and tau reduction due to the longer time to achieve amyloid negativity.

The difference in plasma p-tau dynamics can be explained by the differential pharmacology of the amyloid antibodies. In our amyloid QSP model, we do not discriminate between regular Abeta and N3pE in forming plaques. Donanemab targets the N3pE epitope of Abeta, and reduces SUVR very fast by predominantly removing insoluble plaques that are at the very end of the aggregation cascade. In contrast, because lecanemab and aducanumab to a lesser degree, have a greater effect on reducing intermediate soluble oligomers and protofibrils, they affect neuronal firing more, leading to a more pronounced reduction of tau secretion. This also extends to a new generation of anti-amyloid antibodies currently in clinical trials that are highly specific for soluble oligomers.

When implementing amyloid and tau oligomer effects on ligand- and voltage-gated ion channels, the model predicts a better functional cognitive outcome of amyloid antibody therapy in conditions with a lower pathology and a lower tau burden, especially for an early disease state. This is in line with *post hoc* observations of the clinical effect of lecanemab (Christopher et al., 2023) and to a lesser extent donanemab (Sims et al., 2023).

The basis for this hypothesis stemmed from imaging studies suggesting that in this prodromal AD state, amyloid deposits correspond to metabolic hotspots and hyperactivity to the point that these subjects have a greater probability of suffering from subclinical seizures (Samudra et al., 2023). Both amyloid and tau pathology play a role (Altuna et al., 2022) in epileptogenesis. For example, in brain tissue from refractory epileptic patients, increased amyloid and tau pathology has been found (Gourmaud et al., 2020). In fact, hyperactivity neuropsychiatry symptoms such as disinhibition, irritation and agitation are common in late MCI and early AD (El Haffaf et al., 2024) and are associated with brain morphology in specific brain regions and with regional hyperperfusion (Ferreira and Bastos-Leite, 2024; Billette et al., 2022; Chen et al., 2024; Corriveau-Lecavalier et al., 2021).

The model generates the following hypothesis for this observation. Through their effect on glutamate, α7 nAChR and K^+^ channels, increased soluble amyloid oligomer levels, in early AD and MCI, trigger disinhibition-mediated hyperactivity in the brain. Cognitive tests have suggested that this hyperactivity leads to deficits in specific cognitive domains, which could be alleviated with the anti-seizure medication levetiracetam (Bakker et al., 2012). Other studies however did not find a large degree of hyperactivity (Krebs et al., 2023; Tondo et al., 2022) either due to regional variability or detection limit, suggesting that this increased neuronal firing can be limited. This is in line with our modeling outcomes suggesting that a 5% change in average firing frequency can already lead to substantial changes in ISF, CSF and plasma free tau.

Neuronal firing hyperactivity results in an initial decrease in cognitive capacity, as measured by information capacity in our simulations. This information capacity is proportional to the variability in inter-spike interval. Higher neuronal firing frequency, driven in majority by the refractory period of neuronal firing restricts the range of inter-spike intervals. Studies in human subjects suggest that cognitive performance is not always proportional to neuronal firing but is better associated with statistics of inter-spike interval, as for instance quantified by the concept of information content (Crupi et al., 2018; Dias et al., 2019; Franka et al., 2023; Shew et al., 2011). A recent study suggests variability in BOLDfMRI signal, not absolute intensity, is a potential new biomarker of functional neurological disorders (Schneider et al., 2024). Therefore, reducing neuronal firing frequency can enhance the information bandwidth of the neuronal system and improve cognitive performance as illustrated in Figure 4.

The QSP model suggests that the effect of the soluble oligomers decrease on voltage- and ligand-gated ion channels by different anti-amyloid antibodies leads to a decrease in neuronal firing, counteracting the hyperactivity in these MCI and early AD patients.

The Shannon entropy concept intervals is an efficient way to quantify information based on the variability of inter-spike interval (Rajdl et al., 2017; Souza et al., 2018). By lowering the firing frequency, the anti-amyloid antibodies drive the system from a high firing frequency–where the inter-spike interval is closer to the refractory period–to a lower regimen with a broader distribution of inter-spike intervals. At the same time, this decreases the secretion of tau proteins, likely both in the monomeric and misfolded form.

The model also provides a hypothesis for the observed lack of cognitive benefits in conditions with increased pathology at baseline (i.e., patients more progressed in the disease). This is likely because firing frequency tends to decrease with increasing pathology because of (1) loss of compensatory upregulation of ChAT enzyme that increases cholinergic tone and (2) loss of synapses and neurons as the disease progresses. As a consequence, the beneficial effect of lowering neuronal firing is reduced.

An interesting corollary is the suggestion that this approach could be used to directly simulate the functional effect of therapeutic interventions that block the binding of Abeta oligomers to membrane proteins, such as CT1812 (Rishton et al., 2021).

A similar argument holds when considering tau pathology. The effect of tau oligomers on the strategically positioned Na^+^channels in the Axon Initial Segment, in addition to K^+^ channels dominate any small effect of Abeta oligomers on neuronal activity. This is in line with preclinical observations (Busche, 2019; Busche et al., 2019) and with the fact that cognitive performance in AD patients is related more strongly with tau pathology than with amyloid pathology (Ossenkoppele et al., 2019).

Clinical imaging data suggest that the relation between tau and atrophy is mediated by Abeta positivity in lateral superior temporal and insula and the cognitive function (CDR-SOB) driven by tau-atrophy relationship is mediated by Abeta positivity in rostromedial and paracentral brain region while on average, the tau burden is significantly higher in Abeta positive brain regions (Robinson et al., 2024). Of interest to note is the reduced functional benefit of lecanemab in female subjects (Andrews et al., 2025) in clinical trials. Our analysis suggests that subjects with a higher tau baseline are functionally less responsive to lecanemab therapy, despite a similar reduction in amyloid load. Given that female patients have more tau pathology compared to male patients at the same age (Ossenkoppele et al., 2025) our model could provide a mechanistic interpretation of this gender effect.

The approach in this paper is complementary to a previous model (Mazer et al., 2023) that is based on a semi-empirical model linking amyloid and tau changes to cognitive outcome based on a large number of clinical data. Here we attempt to bring in a mechanistic understanding of the amyloid and tau pathology effects on neuronal firing in a realistic neuronal network that we believe is driving human cognition. This allows us to take into account the pharmacodynamic effects of comedications and common genotypes, such as COMTVal156Met and 5-HTTrs23351 (Geerts and Spiros, 2020b) on the amyloid and tau mediated cognitive outcomes to explain the large variability in functional outcome in clinical trials despite similar effects on amyloid and tau biomarkers.

There are several limitations in this model. First, the aggregation of amyloid species from monomers into plaques in the Interstitial Fluid is very complex and not accessible to intermediate experimental readouts. While certainly not incorporating all the ongoing processes, our model takes into account multiple aspects of the aggregation cascade, including forward and backward reactions and is the most comprehensive of all published amyloid aggregation QSP models (Herriott et al., 2026). It has also predicted a new titration schedule for donanemab that optimizes the amyloid clearance/ARIA side-effects (Geerts et al., 2023a).

Second, the model does not differentiate between different forms of phosphergeorylated tau, as it assumes that all forms of tau are secreted through the same pathway. Recent studies indicate that plasma ptau217 might be a more sensitive biomarker for amyloid pathology (Cecchetti et al., 2024). We assume that unlike total tau, these post-translationally modified tau proteins are derived solely from the brain. While the absolute levels in plasma might differ, the relative normalized decrease from baseline in individual patients is expected to be independent of the tau phosphorylation site. While the QSP model has been calibrated on reported-tau181 changes with the different antibodies, it should be acknowledged that the baseline plasma levels of phosphorylated tau, can be different depending upon the epitope.

Thirdly, the QSP model assumes that human cognition can be simulated by spike train distribution of activity in a cortical microcolumn. While we have previously demonstrated that this is a close proxy for working memory performance–as assessed with a N-back working memory test–in an Alzheimer’s, healthy and schizophrenia environment (Geerts et al., 2013; Roberts et al., 2012), it has not been validated for other aspects of human cognition, such as episodic memory or social functional performance and cognition.

The effect of amyloid and tau oligomers on electrophysiology properties of neuronal circuits is derived from preclinical experiments using rodent or hIPSC based models; with the extrapolation to the human pathology situation not yet demonstrated. Another major issue is the lack of a strong quantitative relationship between levels of Abeta oligomers and their downstream effect on voltage- and ligand-gated ion channels. Many preclinical experiments use unreasonably high concentrations to elicit any detectable effects. Here, by combining the impact of Abeta oligomer dynamics from the QSP model in the lecanemab case with the clinically observed change in plasma p-tau181 using the previously derived relationship between neuronal firing and tau secretion, we can derive a clinically realistic anchor point on the Abeta oligomer strength, i.e., the relation between absolute amyloid oligomer levels and effects on electrophysiological properties.

In contrast, the effects of amyloid plaques on neuron electrophysiological properties are much less studied. One hypothesis starts from the observation that microglia are attracted to plaques *in vivo*, forming a barrier to capture soluble oligomers leaking out of the plaque (Weathered et al., 2024) and in that process secreting pro-inflammatory cytokines. These cytokines have been documented to affect neuronal function, for instance IL10 enhancing VTA DA action (Ronström et al., 2023), while CXCL12 increases excitability in rat amygdala (Sowa et al., 2024). TNFalpha increases excitability (Guo and Lau, 2022), reduces GABA transmission in R47H TREM2 rats (Ren S. et al., 2020; 2021) and is increased in the pre-plaque stage (Cavanagh et al., 2011; Dinet et al., 2022). Other mechanisms include the association of TGFB1 with GABAergic dysfunction in patients (Khedr et al., 2020a; Khedr et al., 2020b)and small-conductance, calcium activated potassium Channel subtype 2 (SK2) channel upregulated after cytokine exposure (Murthy et al., 2015). These processes can be introduced in the QSP model to provide a better prediction of the effect on CDR-SOB of the antibodies that predominantly affect plaques, such as donanemab.

The simulations presented here do not take into account the impact of various comedications (cholinergic medications, antidepressants, benzodiazepines and antipsychotics) on amyloid and tau biomarker and clinical outcome. While there is little evidence that comedications affect the dynamics of amyloid biomarkers after anti-amyloid treatment, they can certainly affect tau secretion through their effect on neuronal firing and certainly clinical outcome (Geerts and Spiros, 2020b). Expanding this approach to simulate virtual twins of real patients that differ with age, gender, amyloid and tau baseline pathology and comedications is beyond the scope of this paper, but will be the subject of a follow-up report.

A major limitation is the absence of a clear link between the tau pathology as measured by PET imaging and the effect of the soluble tau oligomers on Na^+^ and K^+^ channel function. A possible strategy is to explicitly model the aggregation dynamics from tau monomers to oligomers and ultimately to neurofibrillary tangles to derive levels of soluble tau oligomers from Tau-PET imaging studies. Unlike the amyloid aggregation cascade, these processes take place in the neuronal cytoplasm with additional axonal transport and active clearance mechanisms through the autophagy-lysosomal and ubiquitin pathways.

However, the fact that we can semi-qualitatively retrieve the clinical observation of a differential response for anti-amyloid antibodies as a function of baseline disease state and tau load suggests that the QSP model captures a substantial part of the pharmacodynamic effect on functional outcome. Future work will attempt to quantitatively predict changes in CDR-SOB with amyloid or tau therapies when more clinical data becomes available.

The QSP model was previously calibrated with ADAS-Cog clinical data, while the anti-amyloid antibody trials used CDR-SOB as the primary outcome. While reporting on somewhat different clinical symptoms, we have found a tight correlation between close to 300 parallel readouts of these two clinical scales in various patient subgroups. This provides some confidence that the QSP model can also predict CDR-SOB as a clinical readout.

In summary, this report proposes a mechanistic QSP model to better understand the relationship between amyloid and tau dynamics on biomarkers and functional outcomes. Continuously testing its predictions against the clinical data on amyloid therapies in clinical practice might help to identify responders to amyloid and tau therapies on cognitive scales and support identifying optimal trial designs for combination therapy.

## Data Availability

The original contributions presented in the study are included in the article/Supplementary Material, further inquiries can be directed to the corresponding author.
