# Supplementary material for: Understanding quantitative effects of anti-amyloid therapies on tau biomarkers and functional outcome. Insights from a comprehensive mechanistic quantitative systems pharmacology study
Source: Front Pharmacol. 2026 Apr 30;17:1813290. doi: 10.3389/fphar.2026.1813290 (PMC13172624; doi:10.3389/fphar.2026.1813290)
Supplement: Supplementary file 1 [file Supplementaryfile1.docx]

EXPLORING PLASMA P-TAU AS A BIOMARKER FOR ADDRESSING PRACTICAL CHALLENGES IN AMYLOID TREATMENT. A QUANTITATIVE SYSTEMS PHARMACOLOGY STUDY

Hugo Geerts, Shaina Short, Silke Bergeler, Athena Grant, Piet van der Graaf

Certara, Predictive Technologies, USA

SUPPLEMENTARY INFORMATION

# Suppl Section 1. Clinical observations on fluid tau changes and cognitive outcome

The following table lists the observed clinical primary functional Clinical Dementia Rating-Sum of Boxed (CDR-SOB) and Alzheimer’s Disease Assessment Scale (ADAS) score changes and secondary outcomes (plasma p-tau changes) for the different antibodies. These data are used to derive the correlations between the observed readouts and the dynamics of Abeta species from the QSP model.

| **Drug** | **Trial** | **Dose (mpk)** | **Time point (wks)** | **Frequency** | **CDR-SOB Change vs Pla** | **ADAS change vs PLA** | **pTau Reduction (Effect size)** | **Reference** |
| --- | --- | --- | --- | --- | --- | --- | --- | --- |
| LECA | Adaptive Ph2 Trial | 5 | 53 | Q4W |  |  | 0.19 | (McDade et al., 2022) |
| LECA | Adaptive Ph2 Trial | 5 | 53 | Q2W |  |  | 0.35 | (McDade et al., 2022) |
| LECA | Adaptive Ph2 Trial | 10 | 53 | Q4W |  |  | 0.38 | (McDade et al., 2022) |
| LECA | Adaptive Ph2 Trial | 10 | 53 | Q2W |  |  | 0.64 | (McDade et al., 2022) |
| LECA | Adaptive Ph2 Trial | 5 | 78 | Q4W | 0.25 | 0.15 | 0.18 | (McDade et al., 2022) |
| LECA | Adaptive Ph2 Trial | 5 | 78 | Q2W | -0.50 | -2.00 | 0.31 | (McDade et al., 2022) |
| LECA | Adaptive Ph2 Trial | 10 | 78 | Q4W | -0.30 | -0.90 | 0.48 | (McDade et al., 2022) |
| LECA | Adaptive Ph2 Trial | 10 | 78 | Q2W | -0.78 | -2.50 | 0.61 | (McDade et al., 2022) |
| ADU | Low dose EMERGE | 1 - 3 - 6 | 26 | Q4W | -0.09 | -0.23 |  | (Budd Haeberlein et al., 2022) |
| ADU | High dose EMERGE | 1 - 3 - 6 - 10 | 26 | Q4W | -0.13 | -0.47 |  | (Budd Haeberlein et al., 2022) |
| ADU | Low dose ENGAGE | 1 - 3 - 6 | 26 | Q4W | -0.06 | -0.20 |  | (Budd Haeberlein et al., 2022) |
| ADU | High dose ENGAGE | 1 - 3 - 6 - 10 | 26 | Q4W | 0.01 | -0.20 |  | (Budd Haeberlein et al., 2022) |
| ADU | Low dose EMERGE | 1 - 3 - 6 | 78 | Q4W | -0.26 | -0.70 | 0.15 | (Budd Haeberlein et al., 2022) |
| ADU | High dose EMERGE | 1 - 3 - 6 - 10 | 78 | Q4W | -0.39 | -1.40 | 0.27 | (Budd Haeberlein et al., 2022) |
| ADU | Low dose ENGAGE | 1 - 3 - 6 | 78 | Q4W | -0.18 | -0.59 | 0.16 | (Budd Haeberlein et al., 2022) |
| ADU | High dose ENGAGE | 1 - 3 - 6 - 10 | 78 | Q4W | 0.03 | -0.60 | 0.15 | (Budd Haeberlein et al., 2022) |
| DONA | TRAILBLAZER 20mpk | 10 - 20 | 12 | Q4W |  |  | 0.11 | (Pontecorvo et al., 2022) |
| DONA | TRAILBLAZER 20mpk | 10 - 20 | 24 | Q4W |  |  | 0.19 | (Pontecorvo et al., 2022) |
| DONA | TRAILBLAZER 20mpk | 10 - 20 | 36 | Q4W |  |  | 0.21 | (Pontecorvo et al., 2022) |
| DONA | TRAILBLAZER 20mpk | 10 - 20 | 52 | Q4W | 0.35 | 1.05 | 0.22 | (Pontecorvo et al., 2022) |
| DONA | TRAILBLAZER 20mpk | 10 - 20 | 64 | Q4W |  |  | 0.26 | (Pontecorvo et al., 2022) |
| DONA | TRAILBLAZER 20mpk | 10 - 20 | 76 | Q4W | 0.53 | 1.76 | 0.22 | (Pontecorvo et al., 2022) |
| GAN | Scarlett 105 mg | 105 | 104 | Q4W |  |  | 0.03 | (Ostrowitzki et al., 2017) |
| GAN | Scarlett 225 mg | 225 | 24 | Q4W |  |  |  | (Ostrowitzki et al., 2017) |
| GAN | Scarlett 225 mg | 225 | 62 | Q4W |  |  | 0.04 | (Ostrowitzki et al., 2017) |
| GAN | Scarlett 225 mg | 225 | 104 | Q4W |  |  | 0.11 | (Ostrowitzki et al., 2017) |
| GAN | Marguerite -SC 225mg | 225 | 104 | Q4W |  |  | 0.10 | (Ostrowitzki et al., 2017) |
| GAN | DIAN-TU-001-1200mg | 225-1200 | 208 | Q4W | 0.03 |  | 0.19 | (Wang et al., 2022) |
| GAN | DIAN-TU-001-225mg | 225-1200 | 52 | Q4W | 0.02 |  | 0.04 | (Wang et al., 2022) |
| GAN | GRADUATE |  | 114 | Q2W | -0.25 | -1.25 | 0.16 | (Bittner et al., 2022) |
| LECA | CLARITY 10mpk Q2W | 10 | 78 | Q2W | -0.45 | -1.44 | 0.68 | (Christopher H et al., 2023) |
| LECA | CLARITY 10mpk Q2W | 10 | 52 | Q2W | -0.31 | -0.99 | 0.52 | (Christopher H et al., 2023) |
| LECA | CLARITY 10mpk Q2W | 10 | 60 | Q2W | -0.14 | -0.45 |  | (Christopher H et al., 2023) |
| LECA | CLARITY 10mpk Q2W | 10 | 36 | Q2W | -0.07 | -0.24 |  | (Christopher H et al., 2023) |
| LECA | CLARITY 10mpk Q2W | 10 | 24 | Q2W | -0.02 | -0.07 |  | (Christopher H et al., 2023) |
| DONA | TRAILBLAZER ph3 | 10mpk-20mpk | 24 | Q4W | -0.25 | -0.90 | 0.30 | (Sims et al., 2023) |
| DONA |  |  | 52 | Q4W | -0.50 | -1.50 | 0.35 | (Sims et al., 2023) |
| DONA |  |  | 78 | Q4W | -0.66 | -1.52 | 0.34 | (Sims et al., 2023) |
| SOLA | EXPEDITION | 400mg | 80 | Q4W | 0.10 | -1.40 | 0.02 | (Doody et al., 2014) |
| SOLA |  | 100 mg | 52 | QW |  |  |  | (Farlow et al., 2012) |
| SOLA |  | 400 mg | 52 | QW |  |  |  | (Farlow et al., 2012) |
| SOLA |  | 100 mg | 52 | Q4W |  |  |  | (Farlow et al., 2012) |
| SOLA |  | 400 mg | 52 | Q4W |  |  |  | (Farlow et al., 2012) |
| SOLA | EXPEDITION-3 |  | 78 | Q4W | 0.31 | 0.58 |  | (Honig et al., 2018) |
| BAPI 0.15mpk) |  | 0.15 mpk | 78 | Q13W | 0.20 | -0.20 |  | (Salloway et al., 2014) |
| BAPI(0.5 mpk) |  | 0.5mpk | 78 | Q13W | 0.00 | -0.20 |  | (Salloway et al., 2014) |
| BAPI(1mpk) |  | 1mpk | 78 | Q13W | 0.20 | 0.00 |  | (Salloway et al., 2014) |
| BAPI(2mpk) | subcutaneous | 2mpk | 78 | Q4W |  | -0.10 |  | (Brody et al., 2016) |
| BAPI(7mpk) | subcutaneous | 7mpk | 78 | Q4W |  | 0.70 |  | (Brody et al., 2016) |
| BAPI(20mpk) | subcutaneous | 20mpk | 78 | Q4W |  | -1.00 |  | (Brody et al., 2016) |
| ADU | Phase 2 | 3 | 26 | Q4W | 0.20 |  |  | (Sevigny et al., 2016) |
| ADU | Phase 2 | 6 | 26 | Q4W | 0.00 |  |  | (Sevigny et al., 2016) |
| ADU | Phase 2 | 10 | 26 | Q4W | 0.10 |  |  | (Sevigny et al., 2016) |
| ADU | Phase 2 | 3 | 52 | Q4W | -0.50 |  |  | (Sevigny et al., 2016) |
| ADU | Phase 2 | 6 | 52 | Q4W | -0.70 |  |  | (Sevigny et al., 2016) |
| ADU | Phase 2 | 10 | 52 | Q4W | -0.95 |  |  | (Sevigny et al., 2016) |
| SOLA | DIAN-TU | 400/1600 | 204 | Q4W | 0.20 |  | 0.03 | (Wang et al., 2022) |
| SOLA | DIAN-TU | 400/1600 | 52 | Q4W | 0.05 |  | 0.00 | (Wang et al., 2022) |
| CRE | CREAD | 60mpk | 52 | Q4W |  |  | -0.03 | (Ostrowitzki et al., 2022) |
| CRE | CREAD | 60mpk | 100 | Q4W | -0.17 | -0.26 | -0.06 | (Ostrowitzki et al., 2022) |

**Table 1.** List of CDR-SOB and ADAS-Cog differences relative to the placebo treatment in the same clinical trial for 7 different antibodies. Also, the relative changes (effect size) of plasma p-tau versus baseline are also reported.

# Supple Section 2. Reaching Amyloid Negativity

We report on the outcomes of a 1000-patient virtual trial treated for 18 months with clinically relevant doses of the three antibodies.

Overall, 82.9, 48.2 and 60.5% of the virtual patients reached amyloid negativity for donanemab, LECAnemab, and aducanumab respectively. Average duration ranges from 0.9 years (donanemab), to 1.09 years (LECAnemab), and 1.58 years for aducanumab. In order to define when to start sampling patients for plasma p-tau the following figure plots the individual times versus their respective baseline values for the three antibodies.
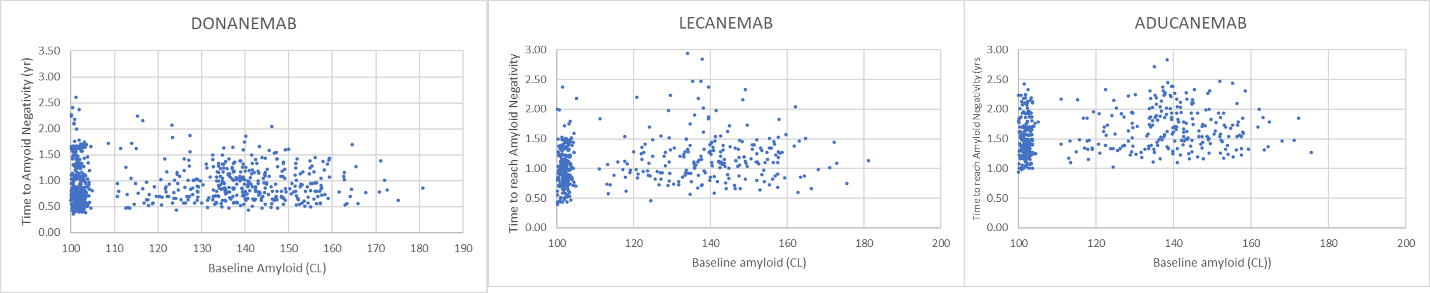


**Figure S1** Times for reaching amyloid negativity as a function of the baseline amyloid load for the virtual patients that reached amyloid negativity (defined as 25CL). This suggests that the earliest time at which to sample for p-tau to derive time of amyloid negativity is 6 months for LECAnemab and 12 months for aducanumab.


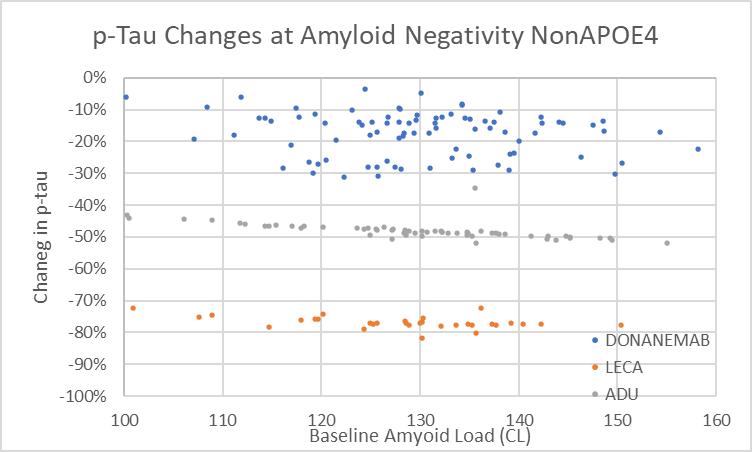


**Figure S2.** Predicted p-tau levels in a virtual patient population of non APOE4 subjects treated with the different antibodies. We retrieve the same results as with the APOE4+ subjects.,

# Suppl section 3. Implementation of the information bandwidth derivation (Information Content)

Here we document the calculation of the Shannon entropy for a specific spike train.

The algorithm for deriving the Shannon entropy of a sike train is as follows

Project spikes times of all neurons on 1 time-axis

K=0 ; *Note index of window-length T*

For time bin [i] =1 to 4 milliseconds

For j=1,4

Window-span ((i-1)*4+j)=4*i*j

*Comment generates the following window-spans : 1*4=4, 1*8=8,1*12=12,1*16=16, 2*4=8, 2*8=16, 2*12=24, 2*16=32, 3*4=12, 3*8-=24, 3*12=36, 3*16=48, 4*4=16, 4*8=32, 4*12=48, 4*16=84 or* ***4,8,12,16,24, 32, 36, 48, 64 milliseconds***

For time spike size =6, 8,10 (seconds starting after stimulus at 2 sec))

Calculate # of words n(i,j, len) with lengths **len** [0,….4*i*j-1] over spike train with ***size***

Calculate probability (normalized to total number of words), i.e. p(i,j,len)=n(i,j,len)/Σ len of {n(i,j)}

Calculate S(i,j,size) = - Σ len of {p(i,j,len) log [p(i,j,len)]}

End size (3 calculations)

Solve A from equations

S(I,j,size1)=A(I,j)+B/size1+C(size1^2)

S(I,j,size2)=A(I,j)+B/size2+C(size2^2)

S(I,j,size3)=A(I,j)+B/size3+C(size3^2)

Intercept_A(k)=A(I,j); Time0(k)=4*i*j

K=k+1

End j (4 loops)

End I (4 loops)

Linear regression Intercept_A (1..9) vs 1/Time0(1…) : *Note some loops above will be redundant because of commutative nature of multiplication i*j*

Intercept_A (k) = α0 +β0/Time0(k)

Entropy = intercept from linear regression = α0.

# Suppl Section 4. Sensitivity analysis Abeta oligomers on neuronal firing and cognition

As outlined in the main text, Abeta oligomers affect various processes, including stimulation of a7 nAChR, AMPA-R on excitatory neurons, GABA-R on interneurons and decreases K+ conductance of pyramidal cells.

Here we report on the effects of those changes associated with Abeta oligomers on firing and information content of the computational neuroscience AD model.


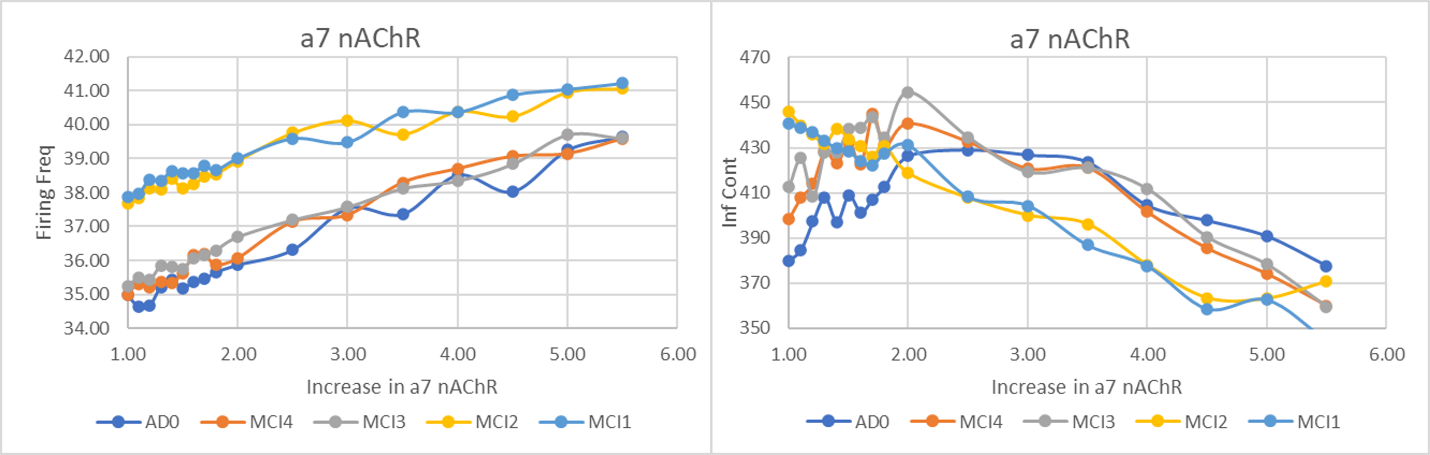


**Figure S3**. Effect of increasing a7 nAChR coupling with NMDA-R on firing frequency (left) and information constant (right) for different conditions ranging from very mild pathology (MCI1) to modest pathology (AD0). While the firing demonstrates a monotonically increasing dose-response, the Information content has the opposite trend for MCI1 and MCI2 and an inverse U-shape trend for MCI3-AD0


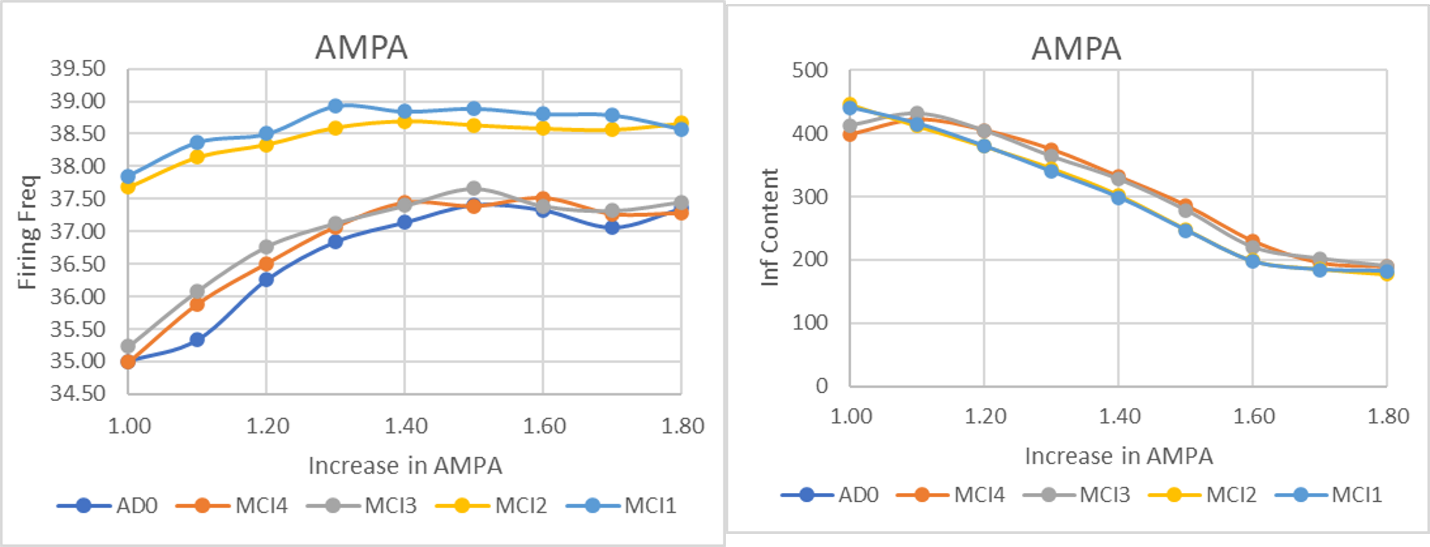


**Figure S4.** Effect of increasing AMPA-R activation on firing frequency (left) and information constant (right) for different conditions ranging from very mild pathology (MCI1) to modest pathology (AD0). While the firing demonstrates a monotonically increasing dose-response, the Information Content has the opposite trend for all conditions.


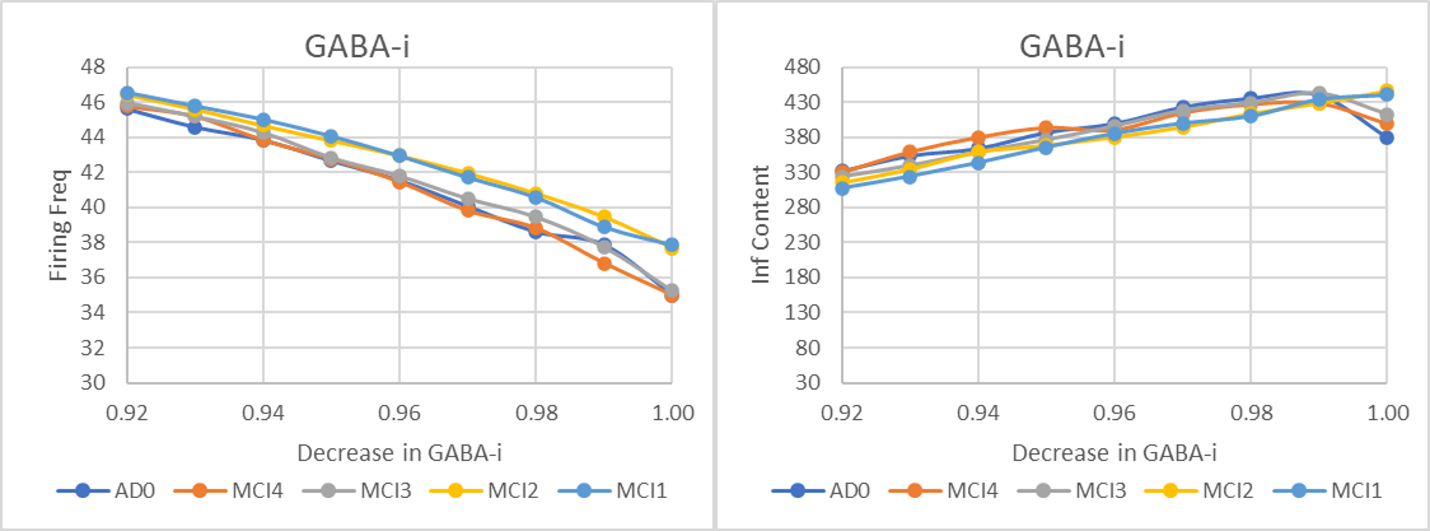


**Figure S5.** Effect of increasing GABA-A receptor activation on interneurons on firing frequency (left) and information constant (right) for different conditions ranging from very mild pathology (MCI1) to modest pathology (AD0). While the firing demonstrates a monotonically decreasing dose-response, the Information Content has the opposite trend for all conditions,


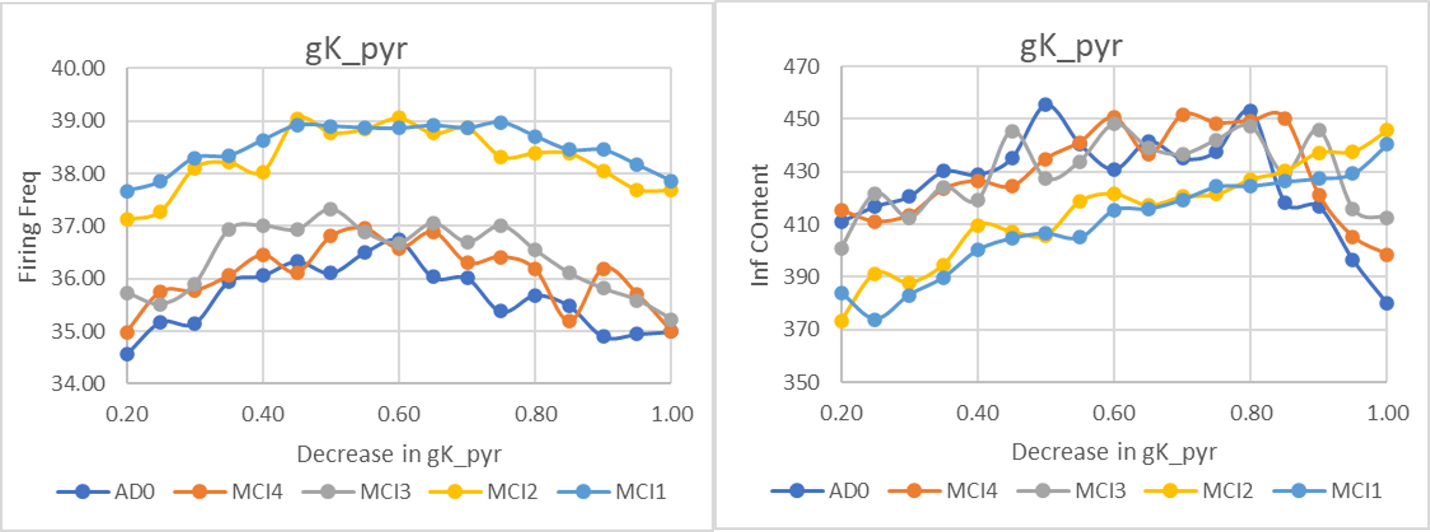


**Figure S6.** Effect of decreasing gK conductance on pyramidal neurons on firing frequency (left) and information constant (right) for different conditions ranging from very mild pathology (MCI1) to modest pathology (AD0). The firing frequency first increases and then decreases as the conductance becomes much smaller. Interestingly, the Information Content decreases monotonically for MCI1 and MCI2 as gK becomes smaller. For MCI3 to AD0 the model demonstrates first an increase followed by a decrease.

**Figure S7** Effect of all Abeta oligomer associated physiological processes on firing frequency (left) and information constant (right) for different conditions ranging from very mild pathology (MCI3) to two levels of modest pathology (AD0 and AD12). The firing frequency increases monotonically, with lower baseline values as the pathology progresses. Interestingly, the information content as defined by the Shannon entropy decreases monotonically for MCI1 and MCI2 with increasing Abeta oligomer strength. For MCI3 to AD0 the model demonstrates first an increase followed by a decrease with the peak shifted to higher Abeta oligomer strength as the disease progresses

# Suppl section 5 – Relationship between neuronal firing and tau secretion

We simulated the preclinical experiments where various pharmacological interventions have been associated with changes in extracellular tau (Yamada et al., 2014) using our single cortical microcircuit Alzheimer model. We investigated three pathology situations (see also (Roberts et al., 2012) )

- heathy controls
- “MCI” case with minimal synapse (3%) and neuronal cell deletion (3%) and hypercholinergic state (75% increase)
- “AD” case with more substantial loss of neurons (5%) and synapses (5%) and a hypocholinergic state (50% loss)

Fig. S7 shows a reasonable correlation between the QSP model simulated changes in firing frequencies (x-axis) and the experimental in vivo changes in tau levels (y-axis) for the three different situations.


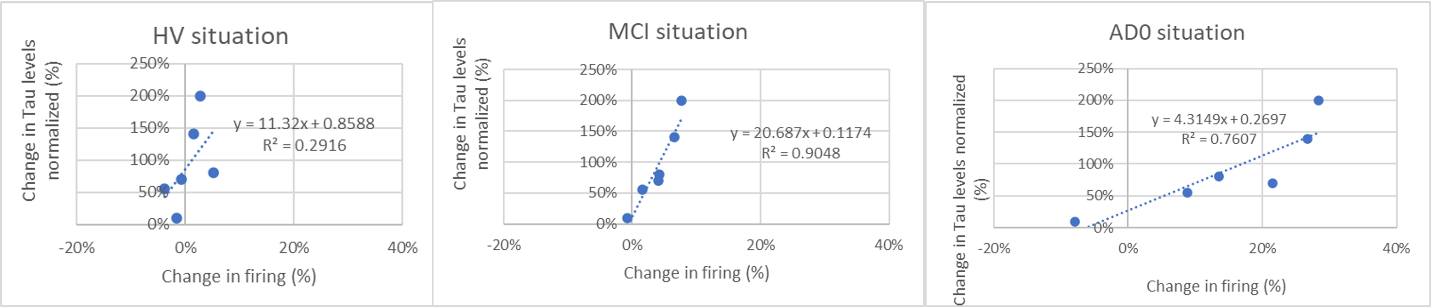


**Figure S7.** Correlation between changes in simulated firing frequencies for three different situations and experimentally observed changes in tau level in wild-type and transgene mouse models.

We calculate a weighted average for the slopes of the 3 conditions, such that 1% of change in firing frequency leads to 12.9 % change in secreted tau.

**Table S2**. lists the experimental observations and corresponding changes in simulated firing frequencies.

| **Intervention** | **Dose** | **Increase in Extracellular-Tau** | **Firing HV** | **Firing MCI** | **Firing AD0** |
| --- | --- | --- | --- | --- | --- |
| Control |  |  |  |  |  |
| Picrotoxin | 25 uM | 200% | 2.75% | 7.70% | 28.43% |
| NMDA | 0.4 uM | 55% | -3.75% | 1.63% | 8.80% |
| NMDA | 4 uM | 70% | -0.72% | 4.07% | 21.47% |
| NMDA | 40 uM | 140% | 1.55% | 6.53% | 26.79% |
| LY | 100 uM | 80% | 5.19% | 4.20% | 13.55% |
| TTX+NMDA | 0.4uM | 10% | -1.50% | -0.71% | -7.92% |
| TTX | 5 uM | 0% | -20.56% | -11.66% | -44.29% |

**Table S2**. Simulated changes in firing frequency with the experimental intervention (column 1) at the dose mentioned (column 2) in the QSP model for a healthy volunteer (column 4), an MCI (column 5) and a mild AD situation (column 6). This allows to link experimentally observed changes in firing frequencies to changes in tau secretion.

Assuming that the plasma p-tau changes reflect the changes in brain ISF tau levels, Table S2 and Fig S3 suggest that the clinically observed decreases in plasma p-tau of 20% for donanemab would correspond to a 2% decrease in firing frequency. Similarly the 40% decrease for aducanumab correspond to a 3.5% decrease in firing frequency and the 60% decrease for LECAnemab corresponds to a 5% decrease in firing frequency.

As shown in Table S3, starting from an Abeta oligomer strength of 10% associated with amyloid positivity in the clinical trials, such decreases in neuronal firing can be achieved by decreases in amyloid strength of 2-8% for donanemab and 5-10% for LECAnemab, depending upon the disease state.

| **amyloid oligo strength** |  |  |  |  |  |  |  |
| --- | --- | --- | --- | --- | --- | --- | --- |
|  | **AD12** | **AD6** | **AD0** | **MCI4** | **MCI3** | **MCI2** | **MCI1** |
| 1 | 87.69% | 92.70% | 96.51% | 95.67% | 95.27% | 97.51% | 97.42% |
| 1.02 | 89.80% | 94.60% | 96.39% | 98.39% | 98.05% | 99.13% | 98.95% |
| 1.04 | 94.24% | 96.19% | 96.39% | 98.76% | 98.65% | 99.20% | 99.63% |
| 1.06 | 96.03% | 97.35% | 101.43% | 101.05% | 100.90% | 100.71% | 100.29% |
| 1.08 | 97.20% | 99.75% | 102.13% | 102.17% | 101.78% | 102.28% | 102.00% |
| 1.1 | 100.00% | 100.00% | 100.00% | 100.00% | 100.00% | 100.00% | 100.00% |
| 1.12 | 101.13% | 102.39% | 104.72% | 105.83% | 104.35% | 103.90% | 99.59% |
| 1.14 | 103.20% | 103.41% | 106.41% | 106.00% | 105.45% | 105.09% | 100.15% |
| 1.16 | 105.01% | 102.91% | 107.06% | 107.38% | 106.74% | 104.76% | 105.36% |
| 1.18 | 106.27% | 104.90% | 108.49% | 108.21% | 107.72% | 106.18% | 105.54% |
| 1.2 | 107.51% | 105.97% | 101.60% | 99.30% | 102.33% | 101.30% | 102.71% |

**Table S3**. Effect of amyloid oligomer interaction on firing frequency in various disease states, assuming that the amyloid pathology increases baseline firing by 10%, over a healthy control situation.

# Suppl section 6 – Functional Outcome with Abeta and tau pathology

Implementing both the physiological impact of Abeta oligomers (section Suppl 4) with the documented effects of tau oligomers mediated decreases in Na+ and K+ channel conductance together with different disease states (ranging from MCI1 to AD12), results in a number of nonlinear relationships as demonstrated in Fig. S7


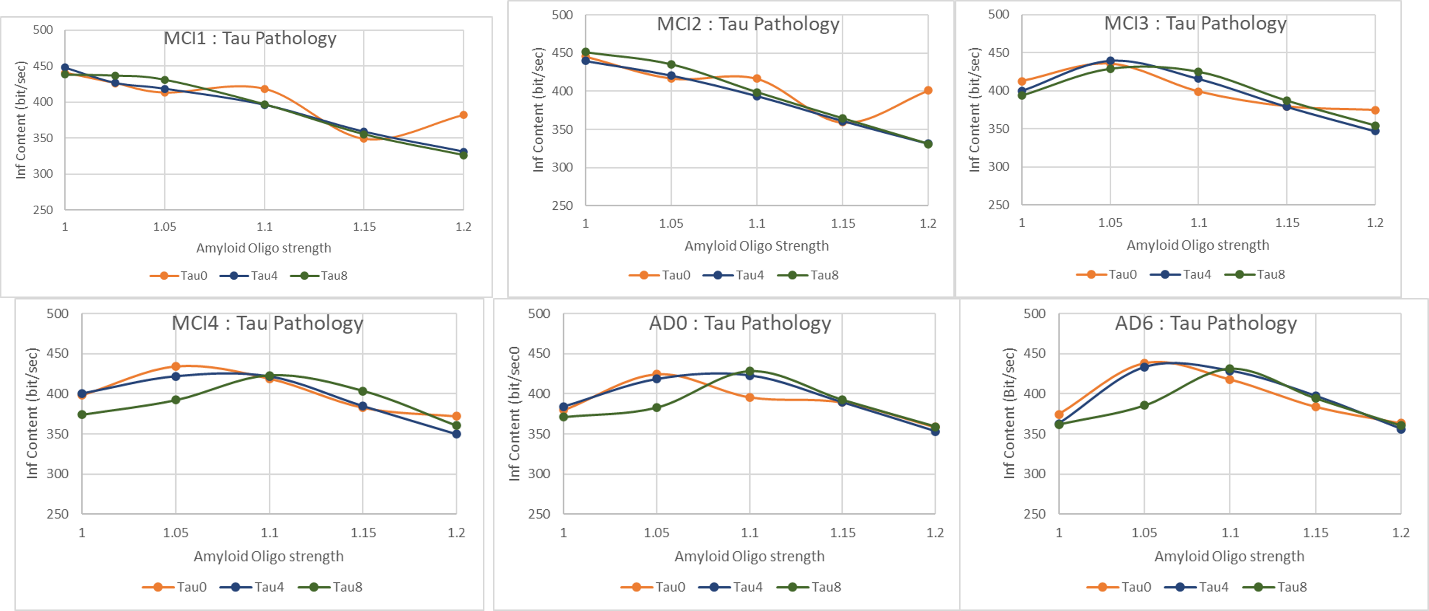


**Figure S8.** Dose-responses of increasing amyloid oligomer pathology on information content in the neuronal QSP model for different disease states (MCI2-AD12) and increasing tau pathology (0=healthy controls, Tau4 = medium tau load, Tau8 = high tau load).

In general, baseline performance decreases with increasing pathology and with increased tau load, most notable in relative advanced pathology. While cognitive performance decreases monotonically with amyloid oligomers at early disease stage (MCI2), an inverse U-shape response becomes apparent with the peak shifting towards higher amyloid oligomer levels as the pathology increases.


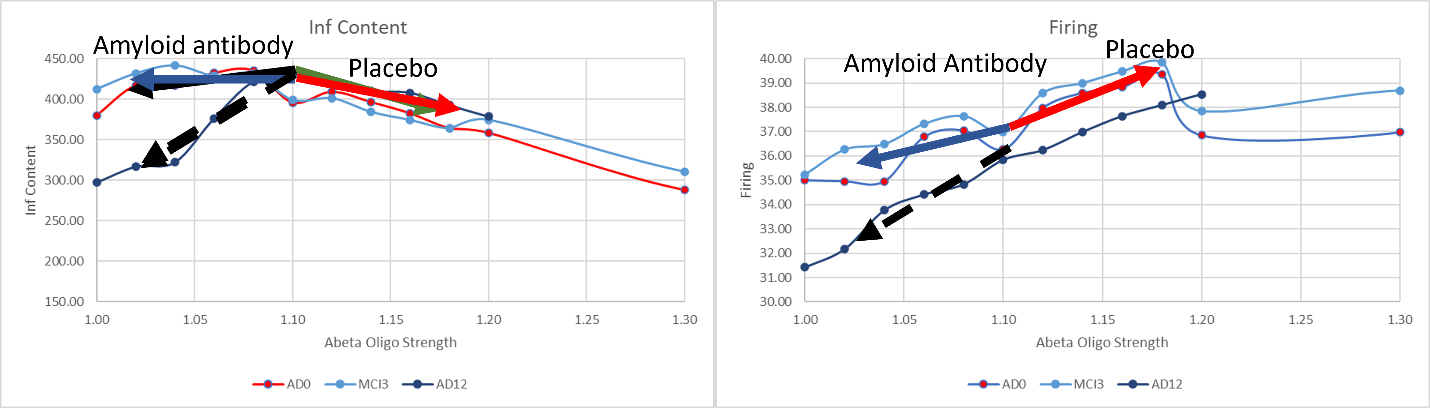


**Figure S9.** Illustration of firing (right) and cognitive (left) trajectories for a patient on treatment (“Amyloid Antibody”) starting from a baseline of Abeta oligomer strength (10%) and reducing by 5%. The blue arrows are for a patient in the MCI3 stage, the stippled black arrow refers to a patient in the AD12 state, while the placebo trajectory (increase in Abeta oligomer strength by 5%) is represented by the red arrow. Comparing blue and black arrows suggest a much greater effect of reducing amyloid load in the MCI3 case as compared to the AD12 case on the information content or cognitive capacity.

Fig S9 depicts a graphical interpretation for the outcome of treatment in early vs more advanced disease state, showing that the cognitive improvement over placebo is much greater in the early disease states.

# Suppl Section 7. Effect of Treatment with amyloid antibodies on CDR-SOB

Here we report on quantitative changes between treated and placebo patients for different scenarios as per Fig. 7 in the manuscript

For a situation (Fig. 7A) where the pathology increases by 1 unit (no change in tau load), differences between active treatment and placebo for an MCI2 baseline pathology are -0.74, -0.64 and -0.27 for low, medium, and high tau. For a disease state of MCI4, the values are -0.57, -0.35, and 0.53 and for AD6 -0.40, -0.06, and 0.48 (negative values reflect better scores for amyloid therapies).

For a situation (Fig. 7C) with an increase for both pathology and tau load, differences are -0.63, -074, and -0.53 for an MCI2 baseline for low, medium and high tau. For a MCI4 baseline state, the values are -0.84, -0.65, and -0.74 and for AD6 -0.17, -0.05, and 0.35 (negative values reflect better scores for amyloid therapies).

Finally, for a situation (Fig. 7D) where only tau load increases by 1 unit, differences are -1.17, -1.21, and -0.27 for an MCI2 baseline for low, medium and high tau. For a MCI4 baseline state, the values are -1.09, -0.58, and -0.26 and for AD0 -0.85, -0.72, and -0.16 (negative values reflect better scores for amyloid therapies).

# Suppl Section 8. Relationship between ADAS-Cog and CDR SOB

Because the original Alzheimer model was calibrated using the ADAS-Cog we investigated whether the model readout would correspond to the more recently used CRD-SOB. To this end, we used Certara’s CODEX database covering 181 clinical studies with ADAS-Cog and 95 studies with CDR-SOB as primary readout and filtered out parallel readings for both clinical scales. This led to 298 data points comparing ADAS-Cog with CDR-SOB for the same patient population.

Fig. S9 shows the correlation between these two scales.


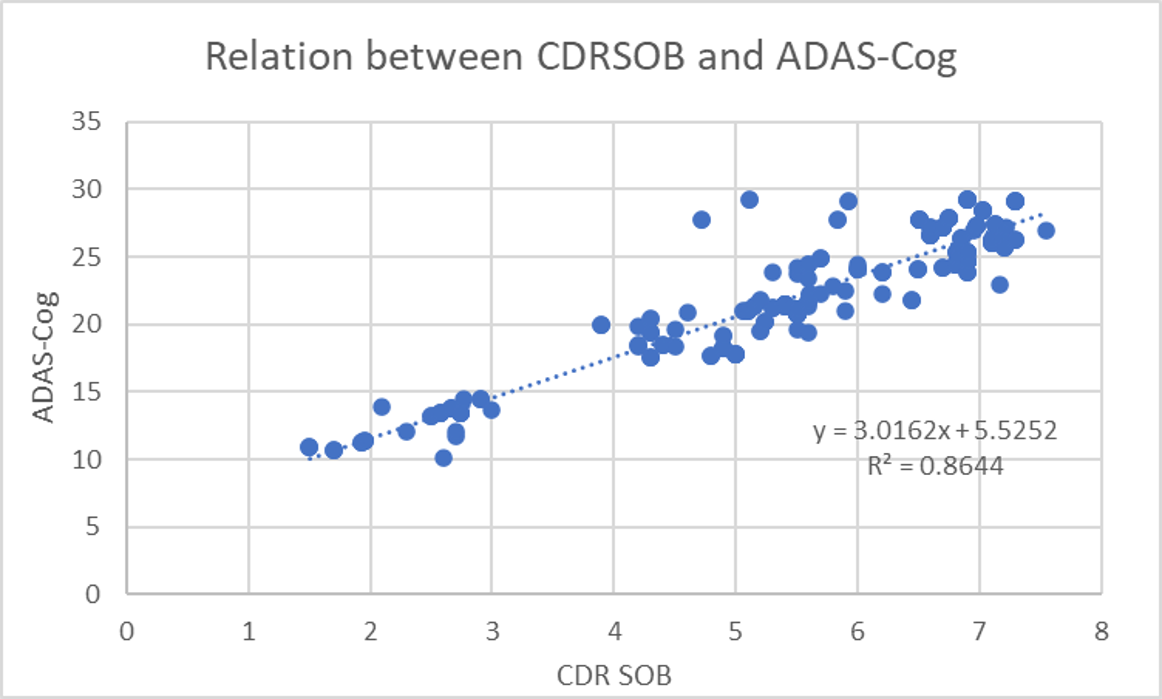


**Figure S10.** Relation between ADAS-Cog and CDR-SOB for 298 data points in clinical readouts demonstrating a high correlation. The equation is then used to translate the QSP model generated simulated ADAS-Cog outcomes to the predicted CDR-SOB outcomes.

References

Bittner, T., Scelsi, M. A., Kollmorgen, G., Jethwa, A., Kerchner, G. A., Fontoura, P., Baudler, M., & Doody, R. S. (2022). Gantenerumab treatment increases plasma beta‐amyloid(1–42) and decreases plasma pTau. *Alzheimer’s & Dementia*, *18*(S5). https://doi.org/10.1002/alz.065684

Brody, M., Liu, E., Di, J., Lu, M., Margolin, R. A., Werth, J. L., Booth, K., Shadman, A., Brashear, H. R., & Novak, G. (2016). A Phase II, Randomized, Double-Blind, Placebo-Controlled Study of Safety, Pharmacokinetics, and Biomarker Results of Subcutaneous Bapineuzumab in Patients with mild to moderate Alzheimer’s disease. *Journal of Alzheimer’s Disease*, *54*(4). https://doi.org/10.3233/JAD-160369

Budd Haeberlein, S., Aisen, P. S., Barkhof, F., Chalkias, S., Chen, T., Cohen, S., Dent, G., Hansson, O., Harrison, K., von Hehn, C., Iwatsubo, T., Mallinckrodt, C., Mummery, C. J., Muralidharan, K. K., Nestorov, I., Nisenbaum, L., Rajagovindan, R., Skordos, L., Tian, Y., … Sandrock, A. (2022). Two Randomized Phase 3 Studies of Aducanumab in Early Alzheimer’s Disease. *Journal of Prevention of Alzheimer’s Disease*, *9*(2). https://doi.org/10.14283/jpad.2022.30

Christopher H, van D., Randall, B., Sharon, C., Rob, M., Marwan, S., Michael, I., & Takeshi, I. (2023). *Lecanemab clarity AD: results from a randomised, double-blind phase 3 early Alzheimer’s disease trial*. https://doi.org/10.1136/jnnp-2023-abn.139

Doody, R. S., Thomas, R. G., Farlow, M., Iwatsubo, T., Vellas, B., Joffe, S., Kieburtz, K., Raman, R., Sun, X., Aisen, P. S., Siemers, E., Liu-Seifert, H., & Mohs, R. (2014). Phase 3 Trials of Solanezumab for Mild-to-Moderate Alzheimer’s Disease. *New England Journal of Medicine*, *370*(4). https://doi.org/10.1056/nejmoa1312889

Farlow, M., Arnold, S. E., Van Dyck, C. H., Aisen, P. S., Snider, B. J., Porsteinsson, A. P., Friedrich, S., Dean, R. A., Gonzales, C., Sethuraman, G., Demattos, R. B., Mohs, R., Paul, S. M., & Siemers, E. R. (2012). Safety and biomarker effects of solanezumab in patients with Alzheimer’s disease. *Alzheimer’s and Dementia*, *8*(4). https://doi.org/10.1016/j.jalz.2011.09.224

Honig, L. S., Vellas, B., Woodward, M., Boada, M., Bullock, R., Borrie, M., Hager, K., Andreasen, N., Scarpini, E., Liu-Seifert, H., Case, M., Dean, R. A., Hake, A., Sundell, K., Poole Hoffmann, V., Carlson, C., Khanna, R., Mintun, M., DeMattos, R., … Siemers, E. (2018). Trial of Solanezumab for Mild Dementia Due to Alzheimer’s Disease. *New England Journal of Medicine*, *378*(4). https://doi.org/10.1056/nejmoa1705971

McDade, E., Cummings, J. L., Dhadda, S., Swanson, C. J., Reyderman, L., Kanekiyo, M., Koyama, A., Irizarry, M., Kramer, L. D., & Bateman, R. J. (2022). Lecanemab in patients with early Alzheimer’s disease: detailed results on biomarker, cognitive, and clinical effects from the randomized and open-label extension of the phase 2 proof-of-concept study. *Alzheimer’s Research and Therapy*, *14*(1). https://doi.org/10.1186/s13195-022-01124-2

Ostrowitzki, S., Bittner, T., Sink, K. M., Mackey, H., Rabe, C., Honig, L. S., Cassetta, E., Woodward, M., Boada, M., Van Dyck, C. H., Grimmer, T., Selkoe, D. J., Schneider, A., Blondeau, K., Hu, N., Quartino, A., Clayton, D., Dolton, M., Dang, Y., … Doody, R. S. (2022). Evaluating the Safety and Efficacy of Crenezumab vs Placebo in Adults with Early Alzheimer Disease: Two Phase 3 Randomized Placebo-Controlled Trials. *JAMA Neurology*, *79*(11). https://doi.org/10.1001/jamaneurol.2022.2909

Ostrowitzki, S., Lasser, R. A., Dorflinger, E., Scheltens, P., Barkhof, F., Nikolcheva, T., Ashford, E., Retout, S., Hofmann, C., Delmar, P., Klein, G., Andjelkovic, M., Dubois, B., Boada, M., Blennow, K., Santarelli, L., & Fontoura, P. (2017). A phase III randomized trial of gantenerumab in prodromal Alzheimer’s disease. *Alzheimer’s Research and Therapy*, *9*(1). https://doi.org/10.1186/s13195-017-0318-y

Pontecorvo, M. J., Lu, M., Burnham, S. C., Schade, A. E., Dage, J. L., Shcherbinin, S., Collins, E. C., Sims, J. R., & Mintun, M. A. (2022). Association of Donanemab Treatment With Exploratory Plasma Biomarkers in Early Symptomatic Alzheimer Disease: A Secondary Analysis of the TRAILBLAZER-ALZ Randomized Clinical Trial. *JAMA Neurology*, *79*(12). https://doi.org/10.1001/jamaneurol.2022.3392

Roberts, P. D., Spiros, A., & Geerts, H. (2012). Simulations of symptomatic treatments for Alzheimer’s disease: Computational analysis of pathology and mechanisms of drug action. *Alzheimer’s Research and Therapy*, *4*(6). https://doi.org/10.1186/alzrt153

Salloway, S., Sperling, R., Fox, N. C., Blennow, K., Klunk, W., Raskind, M., Sabbagh, M., Honig, L. S., Porsteinsson, A. P., Ferris, S., Reichert, M., Ketter, N., Nejadnik, B., Guenzler, V., Miloslavsky, M., Wang, D., Lu, Y., Lull, J., Tudor, I. C., … Brashear, H. R. (2014). Two Phase 3 Trials of Bapineuzumab in Mild-to-Moderate Alzheimer’s Disease. *New England Journal of Medicine*, *370*(4). https://doi.org/10.1056/nejmoa1304839

Sevigny, J., Chiao, P., Bussière, T., Weinreb, P. H., Williams, L., Maier, M., Dunstan, R., Salloway, S., Chen, T., Ling, Y., O’Gorman, J., Qian, F., Arastu, M., Li, M., Chollate, S., Brennan, M. S., Quintero-Monzon, O., Scannevin, R. H., Arnold, H. M., … Sandrock, A. (2016). The antibody aducanumab reduces Aβ plaques in Alzheimer’s disease. *Nature*, *537*(7618). https://doi.org/10.1038/nature19323

Sims, J. R., Zimmer, J. A., Evans, C. D., Lu, M., Ardayfio, P., Sparks, J. D., Wessels, A. M., Shcherbinin, S., Wang, H., Monkul Nery, E. S., Collins, E. C., Solomon, P., Salloway, S., Apostolova, L. G., Hansson, O., Ritchie, C., Brooks, D. A., Mintun, M., & Skovronsky, D. M. (2023). Donanemab in Early Symptomatic Alzheimer Disease: The TRAILBLAZER-ALZ 2 Randomized Clinical Trial. *JAMA*, *330*(6). https://doi.org/10.1001/jama.2023.13239

Wang, G., Li, Y., Xiong, C., McDade, E., Clifford, D. B., Mills, S. L., Santacruz, A. M., Aschenbrenner, A. J., Hassenstab, J., Benzinger, T. L. S., Gordon, B. A., Fagan, A. M., Coalier, K. A., Libre-Guerra, J. J., McCullough, A., Joseph-Mathurin, N., Chen, C. D., Mummery, C., Wendelberger, B. A., … Bateman, R. J. (2022). Evaluation of dose-dependent treatment effects after mid-trial dose escalation in biomarker, clinical, and cognitive outcomes for gantenerumab or solanezumab in dominantly inherited Alzheimer’s disease. *Alzheimer’s and Dementia: Diagnosis, Assessment and Disease Monitoring*, *14*(1). https://doi.org/10.1002/dad2.12367

Yamada, K., Holth, J. K., Liao, F., Stewart, F. R., Mahan, T. E., Jiang, H., Cirrito, J. R., Patel, T. K., Hochgräfe, K., Mandelkow, E. M., & Holtzman, D. M. (2014). Neuronal activity regulates extracellular tau in vivo. *Journal of Experimental Medicine*, *211*(3). https://doi.org/10.1084/jem.20131685
